# Thermal Induced Changes in Cuticle and Cortex to Chemically Treated Hair

**DOI:** 10.1002/bip.70071

**Published:** 2025-12-13

**Authors:** C. R. R. C. Lima, A. C. C. Bandeira, T. S. Martins, L. Otubo, C. L. P. Oliveira

**Affiliations:** ^1^ Institute of Physics, University of São Paulo São Paulo São Paulo Brazil; ^2^ Institute of Environmental, Chemical and Pharmaceutical Sciences, Federal University of São Paulo São Paulo São Paulo Brazil; ^3^ Institute for Energy and Nuclear Research São Paulo São Paulo Brazil

## Abstract

The deterioration of the cuticle and cortex hair due to routine cosmetic practices has been identified as a primary factor contributing to undesirable changes in the esthetic qualities of hair. Chemical and physical treatments can cause significant damage to hair fibers. In this study, the damage induced by heating in chemically treated hair subjected to acid straightening, bleaching, and the combination of both treatments is investigated. Previous results published by our group have already clarified certain aspects of the mechanism of action of acid straightening on hair fibers. In this new work, we show other relevant aspects to the hair care area with the aim to respond: how can we assist cosmetic product developers and consumers in understanding the changes caused by the combined use of chemical transformation procedures (specifically acid straightening and bleaching) and exposure to heat? By examining the thermal behavior of chemically treated hair fibers, we shed light on key aspects of both external (through Fourier transform infrared spectroscopy–attenuated total reflectance [FTIR/ATR] and scanning electron microscopy [SEM]) and internal (through ultra‐small angle x‐ray scattering [USAXS], small angle x‐ray scattering [SAXS], and wide‐angle x‐ray scattering [WAXS]) changes. SAXS and WAXS structural analyses provided information on the internal structure and hierarchical organization of hair samples. While these techniques have been widely used to evaluate hair fibers, the effects of heating on their structure have been less explored. We examined the changes in the hierarchical arrangements as the fibers were heated in situ during the x‐ray scattering experiments. It was possible to evaluate the specific regions where heat causes damage to both the cortex and the cuticle of the hair fiber, and the extent of these damages. Furthermore, it was observed that the bleached and straightened fiber undergoes more changes with the use of heat, due to the loss of important surface components, as shown by FTIR and SEM measurements.

## Graphical abstract

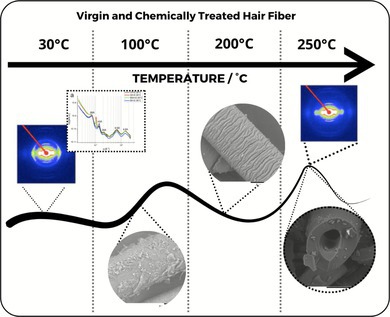

## 1 Introduction

Human hair is composed of three main regions: the cuticle, cortex, and medulla. The outermost layer of the cuticle, the so‐called epicuticle, has a coating of lipids (fatty acids) covalently bonded to the hair surface by thioester links [1, 2]. 18‐methyl eicosanoic acid (18‐MEA) is the predominant component of this lipid layer, playing a crucial role in the protection, lubricity, and hydrophobic properties of the hair [2, 3, 4]. The medulla constitutes the innermost region of the hair [2].

The cortex forms the intermediate layer, organized into macrofibrils, microfibrils, and intermediate filaments (IFs), being the region where most chemical transformations occur. Between the cuticle layers and in the cortex, one has lipidic structures called the cell membrane complex (CMC) [2, 5]. The α‐keratin protein (IFs) defines the mechanical properties of hair, such as strength and elasticity. However, molecular structural alterations in α‐keratin can be utilized to evaluate the effects of cosmetic treatments on hair [6, 7, 8, 9].

Hair α‐keratin undergoes alterations when exposed to cosmetic treatments such as bleaching and acid straightening, as well as thermal processes like flat ironing. These modifications significantly impact the hair's mechanical properties [1, 2, 10]. Some of these treatments may compromise the mechanical properties of hair fibers, leading to reduced tensile strength, increased fragility, and higher porosity. These changes can affect the fiber's water permeability and, in more severe cases, result in irreversible damage [6, 8, 11, 12].

Gamez‐Garcia and Basilan [13] studied the effects of hair blow drying and hot ironing, which induced significant mechanical and thermal stress alterations in the hair. Blow drying elevates the hair temperature to approximately 80°C, leading to rapid water evaporation from the hair fibers. According to the authors, this process generates intense circumferential contraction stresses around the cuticle sheath, which can ultimately result in partial lifting of cuticle cells and the formation of cracks.

The prolonged application of cosmetic treatments such as bleaching and acid straightening on hair fibers promotes the degradation of melanin, detachment of cuticle layers, and the breakdown of 18‐MEA and disulfide bonds [1, 14]. This leads to an increased fragility and porosity of the hair fiber, altering the fiber's water retention mechanisms and resulting in irreversible damage. This progression of damage may also affect the hair's overall appearance and tactile properties (cuticle properties), making it harder to manage and more prone to breakage [3, 4].

Straightening hair treatments have gained widespread popularity in recent decades. In recent years, some compounds, such as glyoxylic acid and its derivatives—including glyoxylic acid combined with carbocysteine and amino acids—have been used in acid‐based formulations for hair straightening and have emerged as an alternative to formaldehyde, which is known to be toxic to human health [14, 15, 16]. However, glyoxylic acid has been recently cited as being possibly responsible for calcium oxalate–induced nephropathy [17]. In order to formalize a set of safe active ingredients for hair straightening, in Brazil, ANVISA (2023) published NI No. 220 of 13/04/2023 (RDC, Resolution of the Collegiate Board, No. 409 of 27/07/2020), the “List of Allowed Actives for Cosmetic Products for Hair Straightening or Waving.” This list specifies the approved straightener ingredients to this moment: thioglycolic acid and its salts, thioglycolic acid esters, sodium or potassium hydroxide, lithium hydroxide, calcium hydroxide combined with guanidine salt, sulfites, and inorganic bisulfites, as well as pyrogallol and thiolactic acid.

Despite this, these acid straighteners are still marketed and used to change the shape of hair permanently in several countries. In addition, few studies have been conducted to understand what these products do to hair and their association with other procedures, such as bleaching and heat tools.

To further study the acid straightening and its association with bleaching and heat tools, we combined multiple experimental techniques, including ultra‐small angle x‐ray scattering (USAXS), small angle x‐ray scattering (SAXS), and wide‐angle x‐ray scattering (WAXS), Fourier transform infrared spectroscopy–attenuated total reflectance (FTIR/ATR), and scanning electron microscopy (SEM). Straightening and bleaching use different mechanisms of action and pHs. While bleaching occurs at around pH 10 (alkaline conditions), acid straightening takes place at around pH 1. This difference in mechanisms and pH, combined with the use of heat from a flat iron (temperature), leads to irreversible damage in the hair.

Additionally, these hair tresses were subjected to in situ experiments associating x‐ray scattering with a temperature‐controlled protocol. These experiments allowed real‐time investigation of crystalline and amorphous structural changes within the hair fibers induced by temperature variations. This research extends our previous investigation [1].

## 2 Materials

### 2.1 Sample Preparation

Untreated Caucasian dark brown hair, sourced commercially from DeMeo Brothers (New York), was fashioned into tresses (2 g and 10 cm long), cleansed at 37.0°C ± 5.0°C with a 10% (w/w) solution of sodium lauryl ether sulfate, and air‐dried for a minimum of 48 h at 22.0°C ± 2.0°C under 55% relative humidity. The hair tresses were subsequently categorized into four groups: virgin/untreated hair (VH), bleached hair (BH), straightened hair (SH), treated with a formulation at pH 1.0 (SH), and bleached and straightened hair treated with a formulation at pH 1.0 (BSH).

### 2.2 Straightened Formulation Preparation (Acid Straightening)

The formulation was prepared as an oil‐in‐water emulsion, comprising the following ingredients according to their International Nomenclature of Cosmetic Ingredients (INCI) names: *Aqua, Behentrimonium Methosulfate (and) Cetearyl Alcohol, Isopropyl Palmitate, PEG‐90M, Polyquaternium‐67, Shea Butter Amidopropyl Trimonium Chloride, Glyoxyloyl Carbocysteine, and Glyoxyloyl Keratin Amino Acids* (15.0%). The pH of the formulation was adjusted to 1.0 using citric acid.

### 2.3 Treatments

#### 2.3.1 Bleaching

The selected hair tresses were chemically damaged through bleaching, using a commercial treatment product containing an alkaline solution (pH 10.5) in an oxidizing medium of hydrogen peroxide (20% [v/v]) and ammonium persulfate, applied for 30 min at room temperature.

#### 2.3.2 Acid Straightening

The tresses were treated with a 1:1 ratio of formulation to hair (1.0 g of formulation per 1.0 g of hair) based on their group classification. The treatment was gently applied to the tresses using a brush to ensure full contact with all the fibers for 20 min. Subsequently, the tresses were brushed, dried with a hairdryer, and straightened 10 times using a flat iron at 180°C.

### 2.4 Methods

#### 2.4.1 X‐Ray Scattering (USAXS, SAXS, and WAXS)

WAXS, SAXS, and USAXS measurements were conducted using a Xeuss 2.0 system from Xenocs, equipped with microfocus GeniX3D sources (Cu Kα, *λ* = 1.54 Å; Mo Kα, *λ* = 0.71 Å; and Cr Kα, *λ* = 2.26 Å), FOX3D collimation optics, and two sets of scatterless slits 2.0. Two‐dimensional scattering intensities were recorded on a PILATUS 300K detector with parameters detailed in Table 1. The data were collected at the EMUSAXS center at the Institute of Physics, University of São Paulo (https://portal.if.usp.br/emu/pt‐br/node/323).

**TABLE 1:** Sources, sample–detector distance values (*D*_sd_) used and their respective ranges for the scattering‐vector values (*q*) used in this work.

		Vector modulus *q* (Å^−1^)	
*D*_sd_ (cm)	Source	*q*_min_	*q*_max_
650 (USAXS)	Cu	0.005	0.040
57.4 (SAXS)	Cu	0.020	0.638
17.3 (WAXS)	Cu	0.085	1.951
17.3 (WAXS)	Mo	0.1924	4.220

X‐ray scattering data were collected in transmission geometry, with all hair fibers carefully aligned parallel to the hair axis. The apparatus used to mount the hair fibers is shown in Figure S1. Around 30 fibers are mounted in the support. Due to anisotropic scattering from the alignment of the hair tresses, sector analysis was performed on the obtained 2D images.

The FIT2D software package [18] was used to carry out azimuthal and radial integrations, generating one‐dimensional curves of scattering intensity as a function of the momentum transfer modulus, *q*, where *q* = 4π/*λ* sin(*θ*), with 2*θ* being the scattering angle. The SUPERSAXS package [19] facilitated standard data treatment procedures as well as promoted the calculation of experimental uncertainties for the scattering data. For the data treatment and background subtraction, the empty cell was used as “blank.”

##### 2.4.1.1 In Situ Temperature‐Variation X‐Ray Scattering Measurements

For x‐ray scattering measurements with temperature variation, a hot‐stage heating sample holder from MRI was used in the Xeuss 2.0 chamber for controlled heating of the sample holder containing the hair fibers. All measurements were performed in vacuum. This sample holder is shown in Figure S2. The heating system includes a control unit, the hot‐stage sample holder, and a chiller, which acts as a thermal reservoir to cool down the heating control unit. X‐ray measurements with heating were conducted at temperatures of 30°C, 50°C, 60°C, 70°C, 80°C, 90°C, 100°C, 110°C, 120°C, 130°C, 140°C, 150°C, 175°C, 200°C, 225°C, 230°C, 245°C, 250°C, 260°C, 270°C, and 300°C. Samples exposed to 30°C, 100°C, 150°C, 200°C, 250°C, and 270°C were also collected and subjected to SEM analysis.

##### 2.4.1.2 Modeling of X‐Ray Scattering Data for Hair Samples

Hair is a complex system composed of a multi‐hierarchical arrangement. In this study, we performed USAXS, SAXS, and WAXS measurements. After various tests, we found out that the best strategy is to model these regions separately. So, the final model for the full scattering intensity of the hair is given by:

(1) $$I_{\mathrm{mod}}\left(q\right)=I_{\text{USAXS}}^{\text{theo}}\left(q\right)+I_{\text{SAXS}}^{\text{theo}}\left(q\right)+I_{\text{WAXS}}^{\text{theo}}\left(q\right)$$

For the USAXS region, a fractal model is capable to provide a reasonable fit, as shown in our previous article [1]:

(2) $$I_{\text{USAXS}}^{\text{theo}}\left(q\right)={S_{C}}_{f}\;S_{\text{fractal}}\left(q\right)\;I_{\text{poly}}\left(q\right)+\text{Back}$$

where $S_{\text{fractal}}\left(q\right)$ is the structure factor for a fractal system (2).

(3) $$S_{\text{fractal}}\left(q\right)=1+\frac{1}{{\left(qR_{0}\right)}^{D_{f}}}\frac{D_{f}\Gamma \left(D_{f}-1\right)}{{\left[1+\frac{1}{\left(q^{2}\xi^{2}\right)}\right]}^{\frac{\left(D_{f}-1\right)}{2}}}\text{sin}\;\left[\left(D_{f}-1\right)\left(\mathrm{q\xi}\right)\;\right]$$

where $I_{\text{poly}}\left(q\right)$ is the form factor of a polydisperse system of spheres with average radius *R*_med_ and polydispersity *σ*.

(4) $$I_{\text{poly}}\left(q\right)=\int_{0}^{\infty }D\left(R_{\mathrm{med}},R,\sigma \right)\cdot R^{6}\cdot I_{\mathrm{sph}}\left(q,R\right)\mathrm{dR}$$

The function $D\left(R_{\mathrm{med}},R,\sigma \right)$ is a number distribution of sizes and is given by a Shulz–Zimm distribution (3). $I_{\mathrm{sph}}\left(q,R\right)$ is the form factor of a solid sphere with radius *R*.

(5) $$I_{\mathrm{sph}}\left(q,R\right)=F_{\mathrm{sph}}^{2}\left[q,R\right]={\left[\frac{3\left(\text{sin}\left(\mathrm{qR}\right)-\mathrm{qR}\;\text{cos}\left(\mathrm{qR}\right)\;\right)}{{\left(\mathrm{qR}\right)}^{3}}\right]}^{2}$$

The parameters for the USAXS modeling are as follows:

*Overall scale parameters

${S_{C}}_{f}$ → overall scale factor

Back → Constant background

*Form factor

$R_{\mathrm{med}}$ → average radius of polydisperse spheres

*σ* → polydispersity

*Structure factor

$D_{f}$ → fractal dimension

ξ → overall fractal size

$R_{0}$ → fractal subunit radius

For SAXS and WAXS, a tentative model combining peak functions and a background was proposed. This approach is similar to previous works in the literature and allows the description of the peak positions and correlation lengths in the hair structure. Given the high complexity of the hair structure, this model is at least capable of describing some structural features of the hair. The theoretical scattering intensity is given by:

(6) $$I_{\mathrm{mod}}\left(q\right)=S_{C}\;S_{\mathrm{agg}}\left(q\right)\;S_{\text{peaks}}\left(q\right)+\text{Back}$$

where *S*_C_ is an overall scale factor, and *Back* is a constant background.

The initial intensity upturn observed on the experimental SAXS curves indicates the presence of large structures inside the hair. These large aggregates are modeled by the inclusion of an aggregate structure factor *S*_agg_(*q*).

(7) $$S_{\mathrm{agg}}\left(q\right)=1+Sc_{\mathrm{agg}}P_{\text{ellip}}\left(q,a,b,c\right)$$

where $Sc_{\mathrm{agg}}$ is the scale factor for the aggregate and *P*_ellip_(*q*) is the form factor of a three‐axial ellipsoid,

(8) $$\begin{array}{c} P_{\text{ellip}}\left(q,a,b,c\right)=1+\frac{2}{\pi }\int_{0}^{\pi /2}\int_{0}^{\pi /2}F_{\mathrm{sph}}^{2}\left[q,r\left(a,b,c,\alpha ,\beta \right)\right]\sin\text{α dα dβ} \end{array}$$

where $\left(a,b,c,\alpha ,\beta \right)={\left[\left(a^{2}\sin^{2}\beta +b^{2}\cos^{2}\beta \right)\sin^{2}\alpha +c^{2}\cos^{2}\alpha \right]}^{1/2}$, and *F*_sph_(*q,r*) is the form factor of a sphere with radius *r*, as shown in Equation (5).

For the description of the internal correlations within the hair a tentative multipeak model was built, based on the strategy described by Forster et al. [20] and Freiberger and Glatter [21]. Similar approaches can be found in the literature for the modeling of scattering data from hair [22].

(9) $$S_{\text{peaks}}\left(q\right)=1+\sum_{i=1}^{N}{S_{C}}_{i}\;\mathrm{Pea}k_{i}\left(q,d_{i},D_{i},\nu \right)$$

The peak shape is obtained by the use of a general versatile function [21],

(10) $$\mathrm{Pea}k_{i}\left(q\right)=\frac{2}{\pi \delta }\frac{\Gamma \left[\nu /2+i\gamma_{\nu }2\left(q-q_{\text{peak}}^{i}\right)/\pi \delta \right]}{\Gamma \left[\nu /2\right]}$$

where

(11) $$q_{\text{peak}}^{i}=2\pi /d_{i}$$

(12) $$\gamma_{\nu }=\pi^{1/2}\frac{\Gamma \left[\left(\nu +1\right)/2\right]}{\Gamma \left[\nu /2\right]}$$

The peak function is normalized such that,$\int_{-\infty }^{\infty }\mathrm{Pea}k_{i}\left(q\right)\mathrm{dq}=1$. The peak width *δ* is related to the domain size *D* by the Debye–Scherrer equation, D=2π/δ. This peak function is convenient since the parameter ν adjusts the peak shape from Lorentzian (ν → 0) to Gaussian (ν → ∞)

(13) $$\left\{\begin{array}{c} \frac{\delta /2\pi }{x^{2}+{\left(\delta /2\right)}^{2}}\;\text{for}\;\nu \to 0\;\left(\text{Lorentzian}\right)\\ \frac{2}{\pi \delta }\exp\left[-\frac{4x^{2}}{\pi \delta^{2}}\right]\;\text{for}\;\nu \to \infty \;\left(\text{Gaussian}\right) \end{array}\right.$$

The parameters on the model are given by:

*Overall scale parameters

$S_{C}$ → overall scale factor

Back → Constant background

*Aggregate

${S_{C}}_{\mathrm{agg}}$ → scale factor for the aggregate

a,b,c → ellipsoid semi‐axis

*Peaks

${S_{C}}_{i}$ → scale factor of peak *i*

$d_{i}$ → correlation length (periodicity)

$D_{i}$ → domain size

ν → peak shape parameter

Typically, three to five peaks are sufficient for a good fit of the scattering data. In this approach, inner‐structure form factors are not included (*P*(*q*) = 1.0) since it is very difficult to include them properly due to the large number of contributions in the system.

The modeling of the USAXS, SAXS, and WAXS regions was performed independently, as the number of parameters for the full fitting would be too large and the modeling would become unstable.

Due to the intrinsic orientation of hair fibers, the corresponding 2D scattering is also oriented. Therefore, following an approach proposed for liquid crystal analysis [23, 24], it is possible to calculate the average order parameter based on the SAXS data. The orientation order is quantified by the orientation distribution function *f*(*β*) of the long molecular axis relative to the director, and the orientation order parameter $\bar{P_{n}}$, defined as follows:

(14) $$\bar{P_{n}}=\int_{0}^{\pi /2}P_{n}\left(\cos\beta \right)f\left(\beta \right)d\left(\cos\beta \right)$$

where *P*_*n*_(*x*) is the *n*th Legendre polynomial. Although the order parameters 𝑃_2_ and can be 𝑃_4_ determined by various experimental methods, the angular function *f* (𝛽) cannot be measured directly. An analytical solution for this integral was proposed by Deutsch [25] based on x‐ray diffraction data and is given by:

(15) $$\bar{P_{2}}=1-\frac{3}{2N}\int_{0}^{\pi /2}I\left(\phi \right)\left\{\sin^{2}\phi +\left(\sin\phi \right)\left(\cos^{2}\phi \right)\ln\left[\left(1+\sin\phi \right)/\cos\phi \right]\right\}\mathrm{d\phi}$$

where the angular origin in this integral is the direction of alignment of the diffraction patterns, and the normalization *N* is defined as follows:

(16) $$N=\int_{0}^{\pi /2}f\left(\beta \right)\left(\sin\beta \right)\mathrm{d\beta}=\int_{0}^{\pi /2}I\left(\phi \right)\mathrm{d\phi}$$

Therefore, the integral over the angular function is substituted by the experimental scattering intensity. For the modeling of the scattering intensity, one has the following equation:

(17) $$\begin{array}{c} I_{\mathrm{OP}}\left(\phi \right)=Sc_{1}L_{\text{peak}}\left(\phi_{1},w,\nu \right)+Sc_{2}L_{\text{peak}}\left(\phi_{1}+180+x_{1},w,\nu \right)+\text{back} \end{array}$$

where $Sc_{1}$ and $Sc_{2}$ are the scale factors for the two peak positions (if available), $\phi_{1}$ is the angular peak position, w is the peak width, ν is the shape factor for the peak (see Equation 10), $x_{1}$ is the correction for the position of the second peak, and Back is a constant background.

The analysis can be performed for one or two peaks, depending on the experimental data. This analysis is important to provide the preferred orientation of the hair fibers in the system. The peak width w is very useful since it provides the angular aperture for the integral over the preferred directions.

#### 2.4.2 Thermogravimetric Analysis (TGA)

TGA measurements were performed using a simultaneous thermal analyzer DSC/TGA, the Discovery SDT 650 from TA Instruments of the Laboratory of Hybrid Materials (LMH), Department of Chemistry, Federal University of São Paulo (UNIFESP). TGA curves were obtained at a heating rate of 10°C min^−1^, in the temperature range from room temperature to 900°C, under a dynamic synthetic air and N_2_ (VH, BH, SH, and BSH) atmosphere (50 mL min^−1^), using a ceramic crucible (90 μL) and a sample mass of around 6 mg.

#### 2.4.3 FTIR/ATR Analysis

The hair tresses (in triplicate) were cut into small fractions and subjected to FTIR spectroscopy. The infrared spectra of hair fibers were recorded in the 400–4000 cm^−1^ range using an Agilent Cary 630 FTIR spectrometer of Max Planck Laboratory (LAMP). IR measurements were performed in ATR mode. All the measurements of FTIR were performed at the Department of Chemistry, Federal University of São Paulo (UNIFESP), with the support of Prof. Tereza da Silva Martins and her group.

#### 2.4.4 SEM Images

##### 2.4.4.1 Caucasian Dark Brown Hair (VH, BH, SH, and BSH)

The surface images of the hair samples were investigated on a table‐top instrument, TM‐3000 Hitachi (15 kV) SEM with tungsten filament and operating at reduced pressure. The hair samples were glued to specific supports for SEM using copper tape and then coated with a layer of 2 nm of Au. All the measurements of SEM were performed at the Laboratory of Microscopy and Microanalysis (LMM) in IPEN São Paulo, Brazil, under the supervision of Dr. Larissa Otubo and her group.

## 3 Results and Discussion

### 3.1 In Situ X‐Ray Scattering Measurements for Hair Tresses Subjected to Temperature‐Controlled Program

USAXS, SAXS, and WAXS patterns were obtained to evaluate changes in the nanostructure of the hair (e.g., IFs and CMC) due to heating. Hair samples before and after cosmetic treatments such as bleaching, acid straightening (and their combination) were subjected to heating.

For the determination of the correct angular slices, radial integrations were performed on the 2D images, and the fitting indicated in Section 2.4.1.2 (Equation 17) was performed. As a result, the fiber orientation and angular aperture for the slices were obtained, allowing correct integrations. These results are shown in Figures S11–S26.

The modeling results of the equatorial and meridional slices using Equations (2) and (6) are fully presented in Figures S27–S59.

#### 3.1.1 Virgin Hair (VH)

**FIGURE 1:**
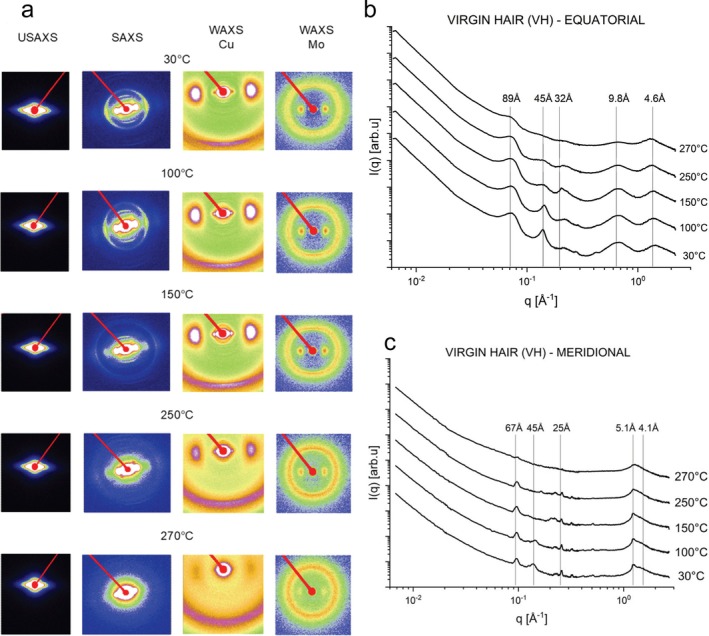
(a) 2D images of USAXS, SAXS, WAXS—Cu and WAXS—Mo of virgin hair at temperatures: 30°C, 100°C, 150°C, 250°C, and 270°C. USAXS, SAXS, and WAXS curves in the equatorial (b) and meridional (c) cuts (section) of virgin hair at temperatures: 30°C, 100°C, 150°C, 250°C, and 270°C.

**FIGURE 2:**
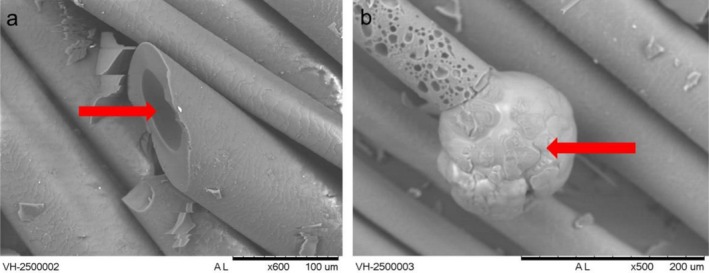
SEM images of virgin hair (VH) tresses at 250°C. Details to (a) the cortex degraded and the cuticle intact, and (b) the cortex melting out of the cuticle.

Figure 1a presents 2D images from USAXS, SAXS, and WAXS scattering of the evolution of behavior of the Caucasian hair fiber under heating for 20 min at 30°C, 100°C, 150°C, 250°C, and 270°C, in situ x‐ray measurements. The angular sectors of the 2D images were taken and combined into single 1D curves as presented in Figure 1b,c. Figure 2b shows the scattering across the fiber (equatorial direction) and Figure 1c along the fiber (meridional direction) for virgin hair (VH) samples. The main signs resulting from the spreading of USAXS, SAXS, and WAXS on the VH fibers are shown in Figure S3.

The peaks in the SAXS region, around *q* ~ 0.071 Å^−1^ (89 Å—equatorial direction) and *q* ~ 0.093 Å^−1^ (67 Å—meridional direction), showed a clear decrease in their intensities after 260°C (see Figure S3). In the 220°C–250°C range, the literature shows an occurrence of several thermal degradations in the cortex, more precisely at the keratin chains, such as melting and denaturation of the alpha‐helix (IFs) and pyrolysis of the amorphous matrix [26, 27, 28].

Figure 2 shows the SEM imaging of the VH tresses at 250°C, which clearly indicates this phenomenon, already reported in the literature [29]. This indicated that the cortex was degrading, decreasing the SAXS peak heights at 89 Å (equatorial) and 67 Å (meridional), both associated with IFs. Baias et al. [30] investigated the phenomenon of thermal denaturation of wool keratin (very similar to hair keratin) in deuterated water by ^1^H NMR and DSC. The authors reported that the mechanism of thermal denaturation of keratin chains follows several steps, with an increase in temperature leading to the breakdown of the IF structure, promoting a metastable state.

According to Istrate et al. [31], although both the cortex (which consists of 21%–22% crystalline IFs) and cuticle (amorphous cross‐linked material) are composed of similar keratin chains, their distinct morphologies result in different thermal stabilities for each component (cortex melts and evaporates above 230°C, and the cuticle remains stable above 250°C). This phenomenon can also be observed in all chemically treated hair samples (BH, SH, and BSH) (see Figures S4–S6).

Figures S7–S10 show the SEM imaging of the evolution of the degradation of the hair samples at 100°C (Figure S7), 200°C (Figure S8), and 250°C (Figures S9 and S10).

The scattering intensity provided by the lipid ring at *q* ~ 0.14 Å^−1^ (45 Å) shows a decrease in intensity and broadening (Figure 1b), suggesting an alteration in the ordering of the CMC [32, 33]. Interestingly, this peak becomes broader at 130°C (see Figure S3a), until it disappears after 260°C. Deeper discussions are presented later in the text. Wade et al. [34] evaluated the changes caused by humidity in hair using SAXS/WAXS measurements. The authors observed that the lipid ring signal for 45 Å appears weaker in some types of hair than others and attributed this to a difference in the lipid content of each type, contributing to the weaker diffraction for the CMC ring [34]. Coderch et al. [33] evaluated the lamellar rearrangement of internal lipids from hair by SAXS, thermogravimetry (TG), and differential scanning calorimetry (DSC). The authors demonstrated that the distance between lipid bilayers is dependent on the amount of polar lipids and water content. The higher permeability of wet fibers leads to a rise in the disorder of the lipid structure [33]. Consequently, our data suggest that, with heating, the water molecules evaporate, decreasing the spacing between the bilayers after 120°C (Figure 1b). Furthermore, TGA data from [33] exhibited the primary degradation step for the extracted hair lipids at 237°C–247°C, which means that they degrade near the range of pyrolysis, denaturation of alpha‐helix chains, and decomposition of cystine bonds. This fact agrees with the SAXS data obtained in this study, which indicates the disappearance of the peak at 45 Å above at 260°C (SAXS diffractogram in Figure S3a).

The thermal behavior of the hair fiber was described and previously published TG‐MS data [27, 28], corroborating the fact that in this temperature range, the highest levels of sulfur‐containing gases (SCO and H_2_S), CO_2_, and NH_3_ are eliminated, indicating the range where the decomposition of the structures of the fiber occurs. Bertrand et al. [35] used XRD experiments and infrared microscopy to evaluate hair strands from two Egyptian mummies of the Greco‐Roman period. Curiously, the authors showed the preservation of the molecular and supramolecular structure of fibrillar keratins (a sharp axial arc at 5.15 Å and broad equatorial spots centered at 9.8 Å) while the lipid diffraction ring was not observed for these ancient hairs. This suggests that the organized lipid fraction has been destroyed or disorganized during the 2000‐year aging process.

Another interesting finding is that the peak at 45 Å in the meridional direction (along the fiber) disappears at temperatures lower than those in the equatorial direction (cross section), about 110°C (see Figure S3). This behavior is, probably, related to the fact that in the equatorial direction, there is a greater contribution from the cuticle, which degrades at higher temperatures than the cortex [26]. As already mentioned, it was reported that the hair cortex melts and evaporates above 230°C, while the cuticle resists over 250°C.

One can note a peak appearing at *q* ≅ 0.1975 Å^−1^ (32 Å) in the equatorial direction, around 130°C, and shifting to higher *q* values (shorter interplanar distances) after 150°C (Figure 1b). The origin of this ring scattering is not clear, but Bertrand et al. [35] observed the existence of one diffuse ring at 29 Å, strongly intensified when the hair is heated around 80°C–90°C, being weakly observed at room temperature. The authors reported that this ring is related to the resulting secondary less ordered liquid crystalline phase, which is reversible after cooling down the hair sample.

Figures S27–S30 (equatorial cuts) and Figures S31–S35 (meridional cuts) present the modeling of x‐ray scattering data for the specific regions USAXS, SAXS, and WAXS (applied for each peak identified in Figure 1a,b) obtained for the VH samples using Equations (2) and (6) at different temperatures (30°C–300°C). The evolution of the modeling parameters are in agreement with the above discussions.

#### 3.1.2 SH, BH, and BSH Hair

**FIGURE 3:**
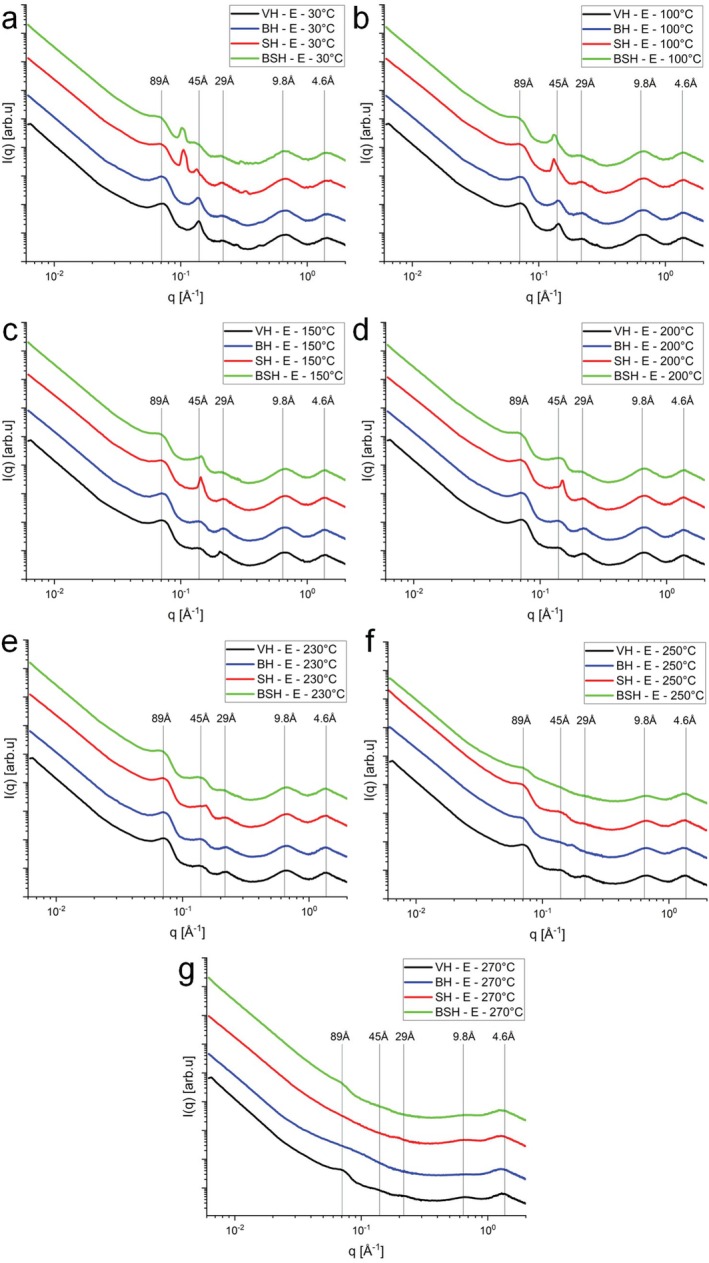
Integrations of the 2D scattering data from equatorial reflections in the SAXS and WAXS zone planes (sample–detector distance: 574 and 173 cm, respectively, Cu source). (a) 30°C, (b) 100°C, (c) 150°C, (d) 200°C, (e) 230°C, (f) 250°C, and (g) 270°C. Virgin hair (VH, in black line), bleached hair (BH, in blue line), straightened hair (SH, in red line), and bleached and straightened hair (BSH, in green line).

**FIGURE 4:**
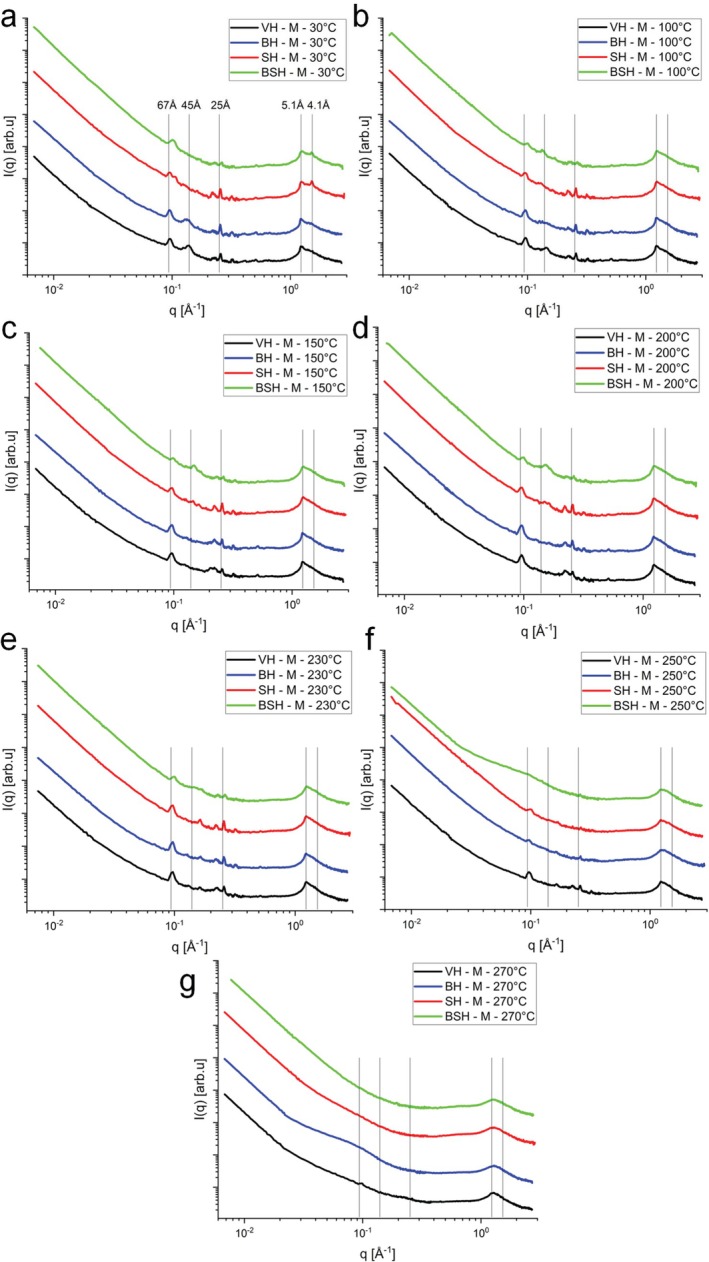
Integrations of the 2D scattering data from meridional reflections in the SAXS and WAXS zone planes (sample–detector distance: 574 and 173 cm, respectively, Cu source). (a) 30°C, (b) 100°C, (c) 150°C, (d) 200°C, (e) 230°C, (f) 250°C, and (g) 270°C. Virgin hair (VH, in black line), bleached hair (BH, in blue line), straightened hair (SH, in red line), and bleached and straightened hair (BSH, in green line).

To explore the effect of the temperature associated with the cosmetic treatments on the protein and lipid structures within the hair samples, the BH, SH and BSH groups were also analyzed using USAXS, SAXS and WAXS experiments under controlled heating at 30°C, 50°C, 60°C, 70°C, 80°C, 90°C, 100°C, 110°C, 120°C, 130°C, 140°C, 150°C, 175°C, 200°C, 225°C, 230°C, 245°C, 250°C, 260°C, 270°C, and 300°C (see Figures S4–S6). In this configuration, it is possible to evaluate different regions in the microstructure of hair as well as investigate length scales between 1 and 4000 Å, that is, almost four orders of magnitude. Figure 3a–g shows the combined integrations obtained from in situ measurements of the SAXS and WAXS regions (at 30°C, 100°C, 150°C, 200°C, 230°C, 250°C, and 270°C) of hair structures in the equatorial direction, and Figure 4a–g in the meridional direction (see Table 1). Similar panels for the treated hair samples are shown in Figures S4–S6.

First, an important observation is the smaller variation in the peak at 89 Å (organization of IFs) of VH compared to chemically treated hair throughout all heating processes, from 30°C to 270°C. This occurs in the equatorial (Figure 3a–g) and meridional (Figure 4a–g) directions. This showed that, one more time, the hair tresses subjected to chemical transformation procedures such as bleaching and straightening become less resistant to temperature [2]. Other studies have shown that procedures such as acid straightening cause changes in the properties of the hair fiber, such as decreased mechanical resistance [16] and increased porosity in the hair fiber when associated with bleaching [1].

Upon heating, the peaks change, similarly to the VH samples. At the SAXS region, three characteristic peaks are clearly identified: *q* ~ 0.07 Å^−1^ (89 Å), *q* ~ 0.14 Å^−1^ (45 Å), and *q* ~ 0.225 Å^−1^ (28 Å). The peak for 89 Å periodicity is less pronounced for the SH tresses (SH and BSH). Interestingly, the peak height of these tresses is similar to VH and BH. Therefore, one can conclude that there is an additional contribution at low *q* values for SH and BSH that is not present for the other samples. As shown in our previous work [1], the acid straightening (using heat flat ironing at 200°C) leads to a significant increase in hair porosity, which affects mainly the low‐angle region. Lima et al. [1] also showed that the porosity can be described by a fractal model, which leads to a characteristic power law and a linear trend in a log × log plot. Therefore, a higher level of porosity leads to an increased contribution to the scattering intensity, which levels off the beginning part of the scattering intensity [1].

Some authors suggest that the cosmetic treatments can alter the IFs of the hair fiber and, consequently, the distances between the microfibrils themselves [36, 37]. This would lead to a change in the peak position, which is not seen in our results. This somewhat corroborates the results from [26], which demonstrated that a decrease in denaturation enthalpy measured by DSC or changes in chemical groups (such as those observed through infrared microscopy by [35]) may suggest alterations to the IFs, but does not necessarily correlate with a loss of crystallinity measured by x‐ray.

Along the meridian axis (Figure 4a–g), one sees the strong and narrow scattering arc at *q* ≅ 0.096 Å^−1^ (67 Å). For the acid‐straightened samples (SH and BSH), one can clearly see the additional contribution from the increased porosity, which levels off the scattering intensity. At 30°C, the SAXS data for VH, BH, and SH show the presence of peaks at 67 Å in the same position and with similar width. However, for the BSH sample, one can see a shift to higher angles and peak broadening, indicating that bleaching combined with acid straightening changes the axial stagger between molecules or groups of molecules along the microfibril, that is, changes the periodic architecture of the molecules along the IFs [8]. The overall behavior remains similar until 200°C. At 250°C, one sees an important loss of ordering for all treated hair tresses, while the structural feature is still visible in the VH (Figure 4b,c). Still along the meridian axis (Figure 4a–g), a small shift to higher *q* values of the peak at *q* ~ 1.230 Å^−1^ (5.10 Å—VH and BH) to *q* ~ 1.234 Å^−1^ (5.09 Å) for the BSH and SH, suggesting a change in the alpha‐helix (along the fiber) structure upon the treatments. This overall trend remains until 200°C.

The peak related to the *q* ~ 1.532 Å^−1^ arc (4.1 Å) becomes more intense and thinner for the acid‐straightened samples (SH and BSH) (Figure 4a). Zhang et al. [38] refer to the arc at 4.1 Å as indicating lipid packing. This suggests that the acid straightening followed by the flat ironing (~180°C) somewhat leads to a local ordering of the lipidic tails along the CMC, which is assumed to be related to this periodicity. Interestingly, this narrow peak disappears already above 70°C (Figures 4b, S5c, and S6c), indicating that it is a metastable induced state. At 250°C and 270°C, one sees a broadening of the 5.1 Å peak, which is expected due to the melting of the structure. This was not observed for the peak at 4.6 Å, related to distances between ß‐sheets [7, 39], which remains at 270°C. This fact corroborates other studies that consider the morphology of the cuticle containing β keratin sheets [27, 40, 41] as being more thermally stable, which was observed in the microscopy images (Figure 2a).

Figures S36–S39 (equatorial cuts) and Figures S40–S43 (meridional cuts) present the modeling of x‐ray scattering data for the specific regions USAXS, SAXS, and WAXS (applied for each peak identified in Figures 3 and 4) obtained for the BH samples using Equations (2) and (6) at different temperatures (30°C–300°C).

Figures S44–S47 (equatorial cuts) and Figures S48–S51 (meridional cuts) present the modeling of x‐ray scattering data for the specific regions USAXS, SAXS, and WAXS (applied for each peak identified in Figures 3 and 4) obtained for the SH samples using Equations (2) and (6) at different temperatures (30°C–300°C).

Figures S52–S55 (equatorial cuts) and Figures S56–S59 (meridional cuts) present the modeling of x‐ray scattering data for the specific regions USAXS, SAXS, and WAXS (applied for each peak identified in Figures 3 and 4) obtained for the BSH samples using Equations (2) and (6) at different temperatures (30°C–300°C). In all cases, the evolution of the modeling parameters are in agreement with the above discussions.

#### 3.1.3 Investigation of Changes in Lipid Ring at 45 Å

**FIGURE 5:**
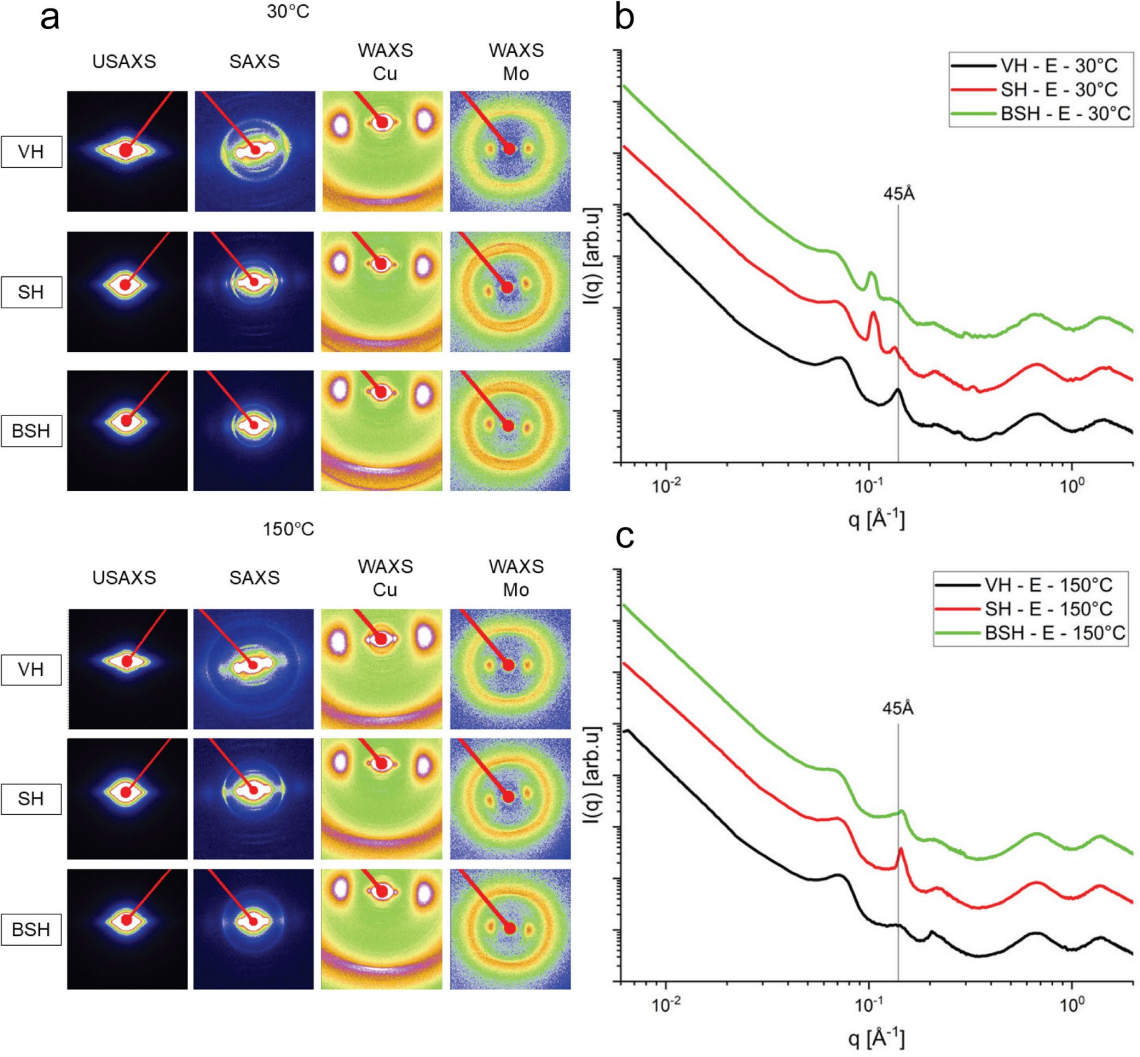
(a) 2D images of USAXS, SAXS, and WAXS zone planes (sample–detector distance: 574 and 173 cm, respectively, Cu source). (b) Equatorial reflection at 30°C, (c) meridional reflection at 150°C. Virgin hair (VH, in black line), straightened hair (SH, in red line), and bleached and straightened hair (BSH, in green line).

The ring for *q* ~ 0.14 Å^−1^ is related to the first peak for the lamellar CMC structure. Along the equatorial direction, Figure 5a–c shows an interesting behavior of the peak around this region. While for the VH and BH one sees a single peak, for the chemically SHs (SH and BSH), one can see two peaks—one for distances of ~45 Å and another for distances of ~59 Å (SH) and ~61 Å (BSH). This fact has already been discussed in the literature, showing that the CMC peaks of the SH and BH hair tresses shifted to lower *q* values, indicating that their lipid structures underwent some type of transformation in the lipid packing [1]. It was concluded that straightening based on the blend of carbonyl‐based compounds associated with flat ironing caused an increase in the lamellar distances by higher retention of water in the CMC due to the formation of the film around the fiber [1].

In this case, it appears that there is a tendency for the crystalline (lipid) planes to orient themselves parallel to the fiber axis. This shift of the peak to lower values of the SH and BSH hair tresses appears to be caused by the increase in the distances between the multilamellar lipid bilayers [13]. This fact leads us to understand that acid straightening causes this change, and this fact may be related to the greater hydrophobicity of this type of chemically transformed hair. To evaluate these changes in SH and BSH hair tresses, we calculated the variations in the periodicity and width of the diffraction peaks related to CMC. For the calculation of the periodicity (*d*_peak_) and crystalline domain size (*D*_peak_), we used Bragg and Debye‐Sherrer laws [42], as mentioned in Section 2.4.1.2. The number of planes is estimated by the ratio D_peak_/d_peak_.

For VH at 30°C, the periodicity is approximately 45 Å (Table 2) and the crystalline domain size is ~340 Å, which corresponds to ~7 planes. However, for the treated hair tresses, BH, SH, and BSH, the CMC peak shifted to around 46 Å (Figure 3a), 59.7 Å (Figure 3a), and 61 Å (Figure 3a), respectively. These data are shown in Table 2.

**TABLE 2:** Comparison between variations in periodicity and crystalline domain between VH, SH, BH, and BSH hair fibers.

Sample	Peak position CMC (*q*_peak_) (${Å}^{-1}$)	CMC peak width (δ) (${Å}^{-1}$)	Periodicity (*d*_peak_) (Å)	Crystalline domain (*D*_peak_) (Å)	Number of plans
VH	0.13788 ± 0.00001	0.0187 ± 0.007	45.57 ± 0.05	340 ± 120	7 ± 3
SH	0.10521 ± 0.00001	0.00533 ± 0.00009	59.72 ± 0.07	1200 ± 20	19 ± 1
BSH	0.10280 ± 0.00004	0.0052 ± 0.0030	61.12 ± 0.21	1200 ± 600	20 ± 10
BH	0.13655 ± 0.00001	0.01554 ± 0.0039	46.01 ± 0.05	400 ± 100	9 ± 2

Interestingly, for BH, the periodicity and crystalline domain size were very close to those of VH, while for the hair fiber subjected to acid straightening (SH and BSH), an increase in the crystalline domain size (~1200 Å) was observed, corresponding to ~20 planes. In other words, the acid straightening process promotes an increase in the size of the crystalline domain, probably due to a greater ordering of the CMC planes that were initially disordered.

As shown in Figures 5b,c, S5b, and S6b, as the temperature increases, the CMC peak of the SH and BSH hair tresses, which were in lower “*q*” positions (higher interplanar distances), shifts to higher *q* values. For values larger than 70°C, the position of the peaks of SH and BSH is already close to that of VH. In fact, Rafik et al. [43] reported that the signals due to lipids are the only variable scattering signals displayed by hair; the signals due to keratin are sample independent. It is important to emphasize that there are no reports in the literature indicating whether this type of cosmetic formulation modifies the interaction of the fiber with other cosmetic products.

### 3.2 TGA

**FIGURE 6:**
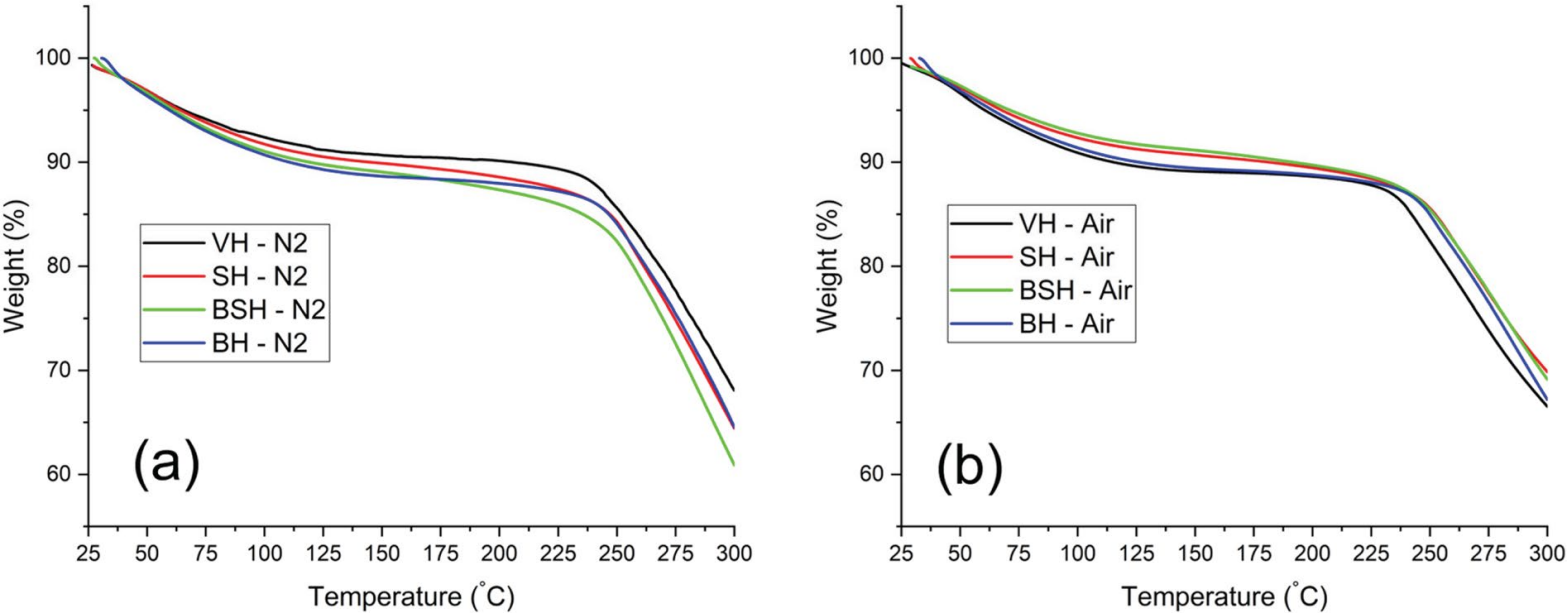
TG/DSC curves obtained at 10°C min^−1^ under dynamic (a) nitrogen (N_2_) and (b) air atmosphere (air); of the samples of hair (virgin and chemically treated hair).

**FIGURE 7:**
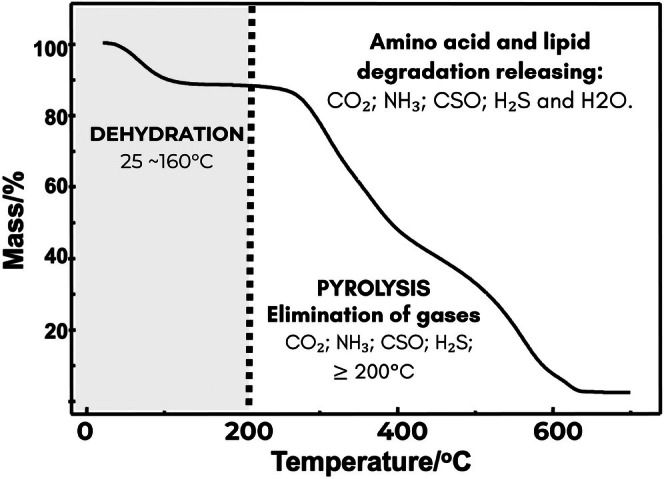
Diagram of the thermal degradation events with mass loss and some gases eliminated during the pyrolysis/denaturation of the hair fiber structures.

TG curves (Figure 6) show the thermal behavior of virgin and chemically treated hair samples within the 25°C–300°C temperature range, under nitrogen (inert) (Figure 6a) and synthetic air (oxidative) (Figure 6b) atmospheres. This temperature range mainly corresponds to dehydration (water loss) and the onset of keratin degradation, accompanied by the release of volatile compounds (CO_2_, H_2_O, SCO, H_2_S, and NH_3_). These processes enable the evaluation of the changes induced by chemical treatments in the thermal stability of hair fibers. Figure 7 presents a diagram showing these thermal degradation events with mass loss and the gases that are eliminated with the pyrolysis/denaturation of the hair fiber structures.

According to the TG results, up to 300°C, all hair types exhibited two distinct mass loss events (Table 3), corresponding to a similar thermal decomposition profile, characterized by (1) dehydration (moisture loss) and (2) subsequent thermal degradation. These observations align with previous findings in the literature [26, 27, 28, 44, 45].

**TABLE 3:** TG results: Range temperature (*ΔT*, in °C) and mass loss (*Δm*, in %) of each event of the hair samples.

Sample	Nitrogen (N_2_)			Synthetic air		
	Dehydration		Decomposition	Dehydration		Decomposition
	*T*_peak_	Mass	*T*_initial_ (°C)	*T*_peak_	Mass	*T*_initial_ (°C)
VH	54	9	210	52	9	200
BH	55	10	210	55	9	205
SH	60	11	200	62	10	175
BSH	56	11	200	55	8	200

Under both atmospheres (N_2_ and air) (Figure 6a), all samples exhibited a predominant mass loss between 30°C and 150°C, attributed to dehydration. A second event occurred from about 210°C (VH and BH) to the hair samples at N_2_, corresponding to the early stages of keratin chain degradation. Comparing these results obtained (Table 3) with those under an oxidative atmosphere (synthetic air), it is observed that the degradation temperature for all samples shifted to lower initial temperatures, which highlights the influence of air on the greater degradation/oxidation of the hair fiber. The results showed changes in the decomposition start temperature for all samples (BH, SH, and especially BSH), specifically, the SH hair samples (*T*_initial_ = 175°C) started decomposition 25°C before the VH samples (*T*_initial_ = 200°C), highlighting the greater damage and decrease in thermal stability of the SH tresses hair.

### 3.3 Alterations on the Chemical Groups of Hair due to Straightening and Bleaching Processes Obtained by FTIR/ATR

**FIGURE 8:**
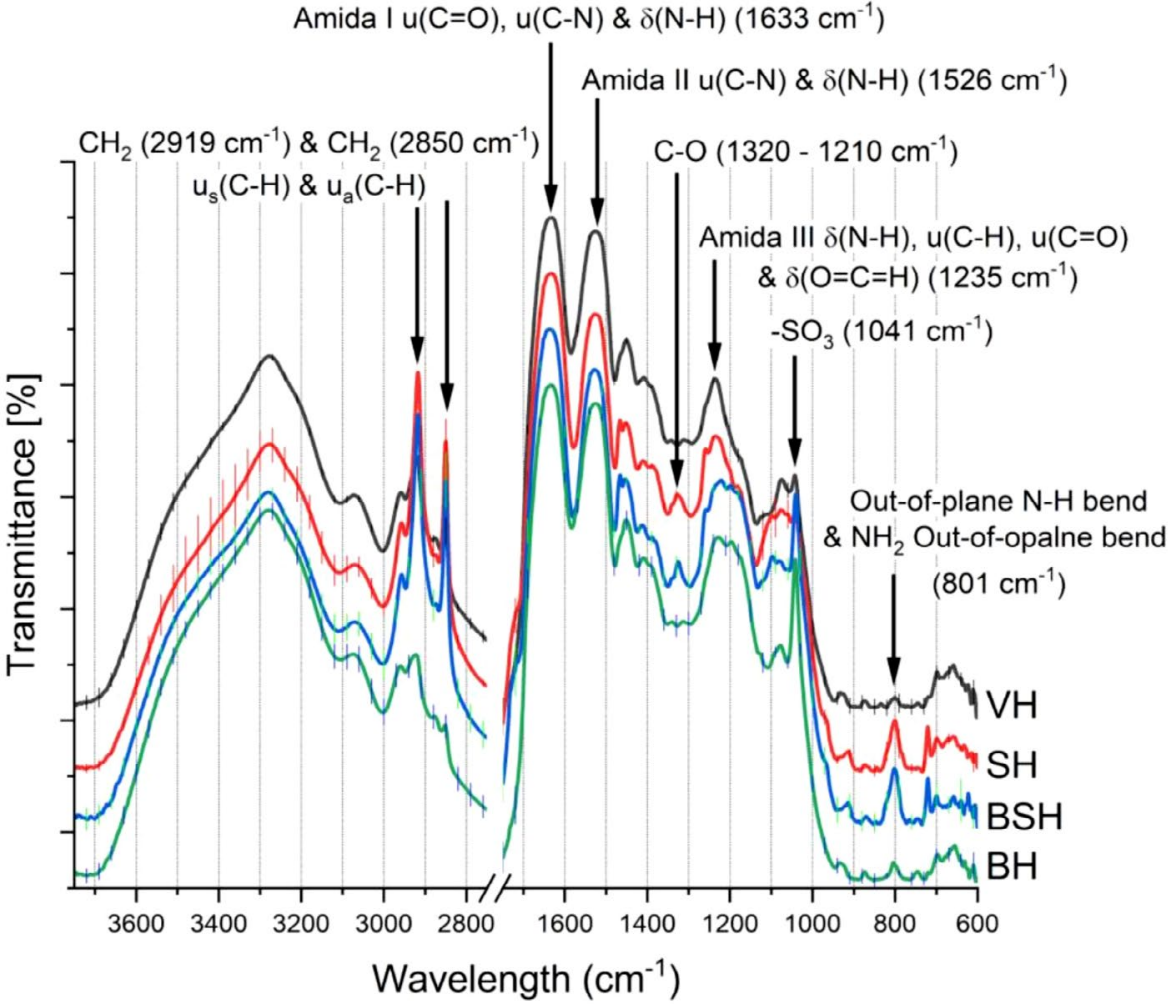
Non‐normalized FTIR/ATR spectra at 500–4000 cm^−1^ comparing virgin and treated hair. The curves were shifted for clarity. Virgin hair (VH, in black line), bleached hair (BH, in green line), straightened hair (SH, in red line), and bleached and straightened hair (BSH, in blue line).

The obtained spectra are characteristic of hair, which is mainly composed of biomolecules such as alpha‐keratin chains, associated proteins, lipids, and other compounds [46]. Even though FTIR/ATR is not a fully quantitative technique, it can give indications about the variation in the lipid levels [32] as well as important changes in the fiber due to the cosmetic treatments. Figure 8 shows the major bands present in the absorption spectra, as well as the average (triplicate) of each hair type sample evaluated. Table 4 presents the main peaks found in our characterization. The obtained spectral data agree with those seen in the literature. Barton [37] studied samples of different types of hair by FTIR/ATR. The author demonstrated that ATR spectra have been obtained with better quality compared to other techniques, allowing the identification of the components of the cuticle and the more peripheral regions of the cortex.

**TABLE 4:** Assignment of the main spectra bands in the hair samples.

Functional group	Wavenumber (cm^−1^)			
	Virgin hair (VH)	Bleached hair (BH)	Straightening hair (SH)	Bleached and straightening hair (BSH)
Amide A	3280	3280	3280	3280
Amide B	3065	3065	3065	3065
CH_2_ Assym	2919	2921	2917	2917
CH_2_ Sym	2850	2850	2850	2850
Amide I	1633	1633	1633	1633
Amide II	1525	1526	1526	1526
Amide II	1450	1450	1450	1450
Amide III	1235	1235	1235	1235
Sulfonic acid	1042	1041	1041	1040
Deformation in NH_2_	801	804	801	802

At 800 cm^−1^, one sees a strong increase in the peaks for SH and BSH, which was promoted to acid straightening in the hair fiber. The primary amines have NH_2_ stretching bands (out‐of‐plane NH_2_) from 850 to 750 cm^−1^ and can involve saturated and aromatic amines. The out‐of‐plane NH_2_ bend involves both hydrogens “wagging” above and below the plane defined by the C–N bond. An out‐of‐plane N–H bend was found at 812 cm^−1^ [47].

Medium‐intensity peaks around 2921 and 2851 cm^−1^ are related to C–H from asymmetric stretching of CH_2_ and symmetric stretching of CH_2_ (modes of lipids from methylene groups) [31, 39, 40]. Reference [39] related the peak at 2852 cm^−1^ to the existence of lipids and fatty acids, indicated by symmetric stretching of the C–H group. Several articles [48, 49] compared the cuticle, cortex, and medulla of human hair based on lipid composition analysis by synchrotron FTIR microspectroscopy. They reported the CH_2_ symmetric stretching peak around 2850 cm^−1^ and CH_2_ asymmetric stretching (near 2921 cm^−1^) as being influenced by lipid organization and long‐range ordering.

**FIGURE 9:**
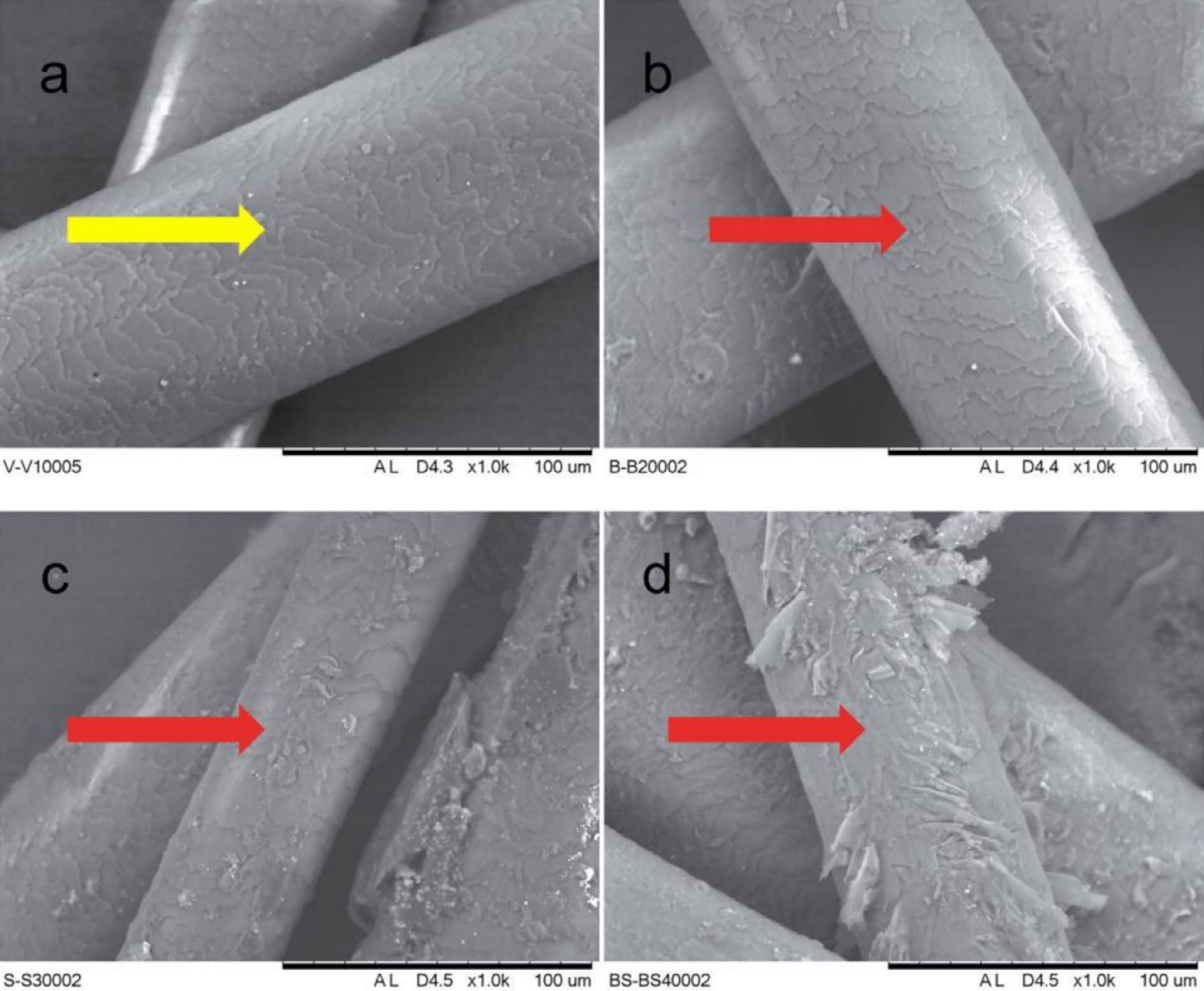
SEM images of virgin (VH; a), bleached (BH; b), straightening (SH; c), and bleached and straightening (BSH; d) at 30°C.

There was an increase in the intensity of the asymmetric and symmetric CH_2_ bands at 2920 and 2851 cm^−1^ signals, respectively [32, 37, 50] to SH (subjected to acid straightening) and BSH (subjected to bleaching and acid straightening) hair samples. The acid straightening associated with heat flat ironing promotes alteration/degradation of the surface lipids, causing irreversible damage to the hair surface. Figures 9d and S7d show some of these alterations.

Analysis of oxidized keratin using infrared instruments has been reported by several authors. Sandt and Borondics [48] showed that while the medulla has elevated lipid levels, the cuticle spectra show an intermediate level of lipids and stronger cysteic acid and sulfonate peaks at 1175 and 1040 cm^−1^ due to the oxidative hair treatment [49]. Bands of residues from the breakdown of cystine bonds were also detected, mainly in chemically treated hair tresses. The intensity of the band at 1042 cm^−1^ (–SO_3_H) increased. It is known that oxidizing agents and thermal treatment disrupt keratin disulfide bonds, forming cysteic acid and other subproducts [12, 27, 37, 50]. These species result in a reduction in keratin structural rigidity and resistance of the hair fiber. Furthermore, the increase in negative charges on the surface of the hair fiber generates an increase in hydrophilicity and consequently in frizz [11, 51, 52]. Korte et al. [53] evaluated the emergence of chemical groups at the surface of hair fibers subjected to bleaching. They found signals of cysteic acid residues by 18‐MEA breaking, enhancing the hair's hydrophilicity. These changes observed in FTIR on the chemical groups in the surface of the fibers suggest that the surface became more jagged and oxidized with the treatments. This can promote cracks or wear on the cuticle, which can be explored by microscopy. The images in Figure 9 show some changes in the surface of the fibers, which show the differences in the surfaces of samples of VH, BH, SH, and BSH.

The yellow arrow indicates the healthy and regular alignment of cuticle layers in the VH. The red arrows indicate regions where the cuticle layers were ripped. Compared to VH, most of the hair cuticle was removed in the case of bleached and straightened hair (BH and SH), indicating a high degree of damage to the hair surface subjected to chemical overlapping treatments (BSH) and heat. With the breakdown of cystine bonds and 18‐MEA breaking in the surface of the hair, the surface is negatively charged and chemically damaged, which causes parts of the scales to fracture and reveal underlying cuticle remnants [2, 49].

A band at 1324 cm^−1^ appeared for the hair samples SH and BSH. The C–O stretch around 1320–1210 cm^−1^ is related to carboxylic acids [54], which can justify the presence of these groups in SH [55]. It is reported that the stretch at 1340 cm^−1^ corresponds to S=O stretching due to sulfonic acid, which also results from the breaking of sulfur bonds (cystine). It is known that acid straightening causes denaturation of the IFs and pyrolysis above 200°C, promoting the decomposition of sulfur‐containing amino acids (including cystine) in the cuticle and/or cortex of the hair, thereby weakening the fiber [1, 27].

The amide III band (VH) displayed a similar line shape and maxima, exhibiting a strong, sharp absorption at approximately 1235 cm^−1^ (N–H bending) [56, 57, 58]. However, the peak maximum position of all treated hair spectra (BH, SH, and BSH) exhibited an alteration in intensity, suggesting a change in protein conformation. According to Kuzuhara [12], an increase in the amide III (unordered) band suggests changes in protein structures to random coil form.

## 4 Conclusions

This study systematically explored the thermal behavior of natural and chemically treated Caucasian hair fiber and correlated it with superficial (cuticle) and internal (cortex). It was possible to analyze alterations in the cuticle (surface region) and cortex (internal region) of hair tresses subjected to chemical treatments and then heating. Combining SAXS and WAXS with SEM, we evaluated the alterations in the microstructures of the hair cortex. Combining FTIR/ATR with SEM, it was possible to identify the chemical groups and surface damage due to multiple cosmetic treatments. Acid straightening promotes alteration of the lipids and proteins, either in the cortex (observed by SAXS and WAXS) or in the cuticle (observed by FTIR/ATR and SEM). Our results showed that beta‐keratin, primarily present in the cuticle, is more resistant to temperature than alpha‐helix chains, supporting the findings in the literature.

It was clearly observed that the hair structure and temperature response are modified when it is subjected to cosmetic treatments, such as bleaching and acid straightening. Our results demonstrate that straightening leads to metastable structural states that are relaxed when subjected to temperatures above 70°C. Therefore, at least from the structural point of view, it is not recommended to expose straightened hair fibers to high temperatures since it compromises the cosmetic treatment. This can influence the temperature used in the hair blower for hair drying procedures.

The combination of the techniques shown in this work allowed the evaluation of the structural, thermodynamic, vibrational, and microscopic properties of hair. Moreover, this work provides new insights into the lipid arrangement within the hair fiber and how the structure is modified by common cosmetic procedures in the beauty industry.

Our study reaffirmed that the use of a multifactorial approach, combining x‐ray scattering methods (USAXS, SAXS, and WAXS) with other analytical methods, such as SEM, DSC, TG, and FTIR/ATR, can provide structural details on a broad range of length scales. These results allow a better understanding of the effects of cosmetic procedures, optimize treatments, and can be used to validate claims made by the hair care industry.

## Funding

The authors were supported by the São Paulo Research Foundation (FAPESP) (grant #2016/24531‐3), the Conselho Nacional de Desenvolvimento Científico e Tecnológico (CNPq) (grants #303001/2019‐4 and #307303/2023‐3), and the Coordenação de Aperfeiçoamento de Pessoal de Nível Superior.

## Conflicts of Interest

The authors declare no conflicts of interest.

## Supporting information


**Data S1:** Supporting Information.

## Data Availability

The data that support the findings of this study are available in the Supporting Information S1 of this article.

## References

[1] C. R. R. C. Lima , R. J. S. Lima , A. C. C. Bandeira , R. A. A. Couto , H. N. Bordallo , and C. L. P. Oliveira , “Alterations Promoted by Acid Straightening and/or Bleaching in Hair Microstructures,” Journal of Applied Crystallography 56 (2023): 1002–1014, 10.1107/S1600576723005599. 37555227 PMC10405601

[2] C. R. Robbins , Chemical and Physical Behavior of Human Hair, 5th ed. (Springer, 2012).

[3] S. Tokunaga , H. Tanamachi , and K. Ishikawa , “Degradation of Hair Surface: Importance of 18‐MEA and Epicuticle,” Cosmetics 6, no. 2 (2019): 31, 10.3390/cosmetics6020031.

[4] E.‐S. Kim , S.‐K. Son , and C.‐K. Lee , “Recovery of Covalently Linked Fatty Acid Monolayer on the Hair Surface Using Biomimetic Lipid,” Journal of the Society of Cosmetic Scientists of Korea 38, no. 2 (2012): 139–145.

[5] B. Bhushan , Biophysics of Human Hair: Structural, Nanomechanical, and Nanotribological Studies (Springer, 2010).

[6] F. J. Wortmann and H. Deutz , “Thermal Analysis of Ortho‐ and Para‐Cortical Cells Isolated From Wool Fibers,” Journal of Applied Polymer Science 68, no. 12 (1998): 1991–1995.

[7] L. Kreplak , J. Doucet , and F. Briki , “Unraveling Double Stranded Alpha‐Helical Coiled Coils: An X‐Ray Diffraction Study on Hard Alpha‐Keratin Fibers,” Biopolymers 58, no. 5 (2001): 526–533, 10.1002/1097-0282(20010415)58:5<526::aid-bip1028>3.0.co;2-l. 11241224

[8] L. Kreplak , A. Franbourg , F. Briki , F. Leroy , D. Dalle , and J. Doucet , “A New Deformation Model of Hard Alpha‐Keratin Fibers at the Nanometer Scale: Implications for Hard Alpha‐Keratin Intermediate Filament Mechanical Properties,” Biophysical Journal 82, no. 4 (2002): 2265–2274, 10.1016/s0006-3495(02)75572-0. 11916881 PMC1302019

[9] J. E. Plowman , D. P. Harland , and S. Deb‐Choudhury , The Hair Fibre: Proteins, Structure and Development (Springer, 2018).

[10] M. V. Robles Velasco , M. V. R. Velasco , T. C. d. S. Dias , et al., “Hair Fiber Characteristics and Methods to Evaluate Hair Physical and Mechanical Properties,” Brazilian Journal of Pharmaceutical Sciences 45, no. 1 (2009): 153–162, 10.1590/s1984-82502009000100019.

[11] A. Kuzuhara , “Analysis of Structural Changes in Bleached Keratin Fibers (Black and White Human Hair) Using Raman Spectroscopy,” Biopolymers 81, no. 6 (2006): 506–514, 10.1002/bip.20453. 16425172

[12] A. Kuzuhara , “Analysis of Structural Change in Keratin Fibers Resulting From Chemical Treatments Using Raman Spectroscopy,” Biopolymers 77, no. 6 (2005): 335–344, 10.1002/bip.20221. 15739182

[13] M. Gamez‐Garcia and J. Basilan , “The Use of Image Analysis to Assess the Role of Polymers on the Thermal Protection of Asian Hair,” 2019.

[14] T. Barreto , F. Weffort , S. Frattini , G. Pinto , P. Damasco , and D. Melo , “Straight to the Point: What Do We Know So Far on Hair Straightening?,” Skin Appendage Disorders 7, no. 4 (2021): 265–271, 10.1159/000514367. 34307473 PMC8280444

[15] J. N. Hatsbach de Paula , F. M. A. Basílio , and F. A. Mulinari‐Brenner , “Efeitos dos alisantes químicos na haste do pelo e no couro cabeludo: revisão,” Anais Brasileiros de Dermatologia (Portuguese) 97, no. 2 (2022): 193–203, 10.1016/j.abdp.2022.01.006. PMC9073307 35058079

[16] A. M. Goshiyama , M. F. Dario , C. Lima , G. L. B. de Araujo , A. R. Baby , and M. V. R. Velasco , “Impact of Acid Straightener's pH Value in the Hair Fiber Properties,” Journal of Cosmetic Dermatology 19, no. 2 (2020): 508–513, 10.1111/jocd.13006. 31241825

[17] T. Robert , E. Tang , J. Kervadec , J. Zaworski , M. Daudon , and E. Letavernier , “Kidney Injury and Hair‐Straightening Products Containing Glyoxylic Acid,” New England Journal of Medicine 390, no. 12 (2024): 1147–1149, 10.1056/NEJMc2400528. 38507759

[18] A. P. Hammersley , “FIT2D: A Multi‐Purpose Data Reduction, Analysis and Visualization Program,” Journal of Applied Crystallography 49 (2016): 646–652, 10.1107/s1600576716000455.

[19] C. L. P. Oliveira , T. Vorup‐Jensen , C. B. F. Andersen , G. R. Andersen , and J. S. Pedersen , “Discovering New Features of Protein Complexes Structures by Small‐Angle X‐Ray Scattering,” in Applications of Synchrotron Light to Scattering and Diffraction in Materials and Life Sciences, ed. M. Gomez , A. Nogales , M. C. Garcia‐Gutierrez , and T. A. Ezquerra (Springer, 2009), 231–244.

[20] S. Forster , A. Timmann , M. Konrad , et al., “Scattering Curves of Ordered Mesoscopic Materials,” Journal of Physical Chemistry B 109, no. 4 (2005): 1347–1360, 10.1021/jp0467494. 16851102

[21] N. Freiberger and O. Glatter , “Small‐Angle Scattering From Hexagonal Liquid Crystals,” Journal of Physical Chemistry B 110, no. 30 (2006): 14719–14727, 10.1021/jp0559332. 16869579

[22] N. S. Murthy , W. J. Wang , and Y. Kamath , “Structure of Intermediate Filament Assembly in Hair Deduced From Hydration Studies Using Small‐Angle Neutron Scattering,” Journal of Structural Biology 206, no. 3 (2019): 295–304, 10.1016/j.jsb.2019.04.004. 30951823

[23] O. R. Santos , D. Reis , A. G. Oliveira , C. L. P. Oliveira , and A. M. F. Neto , “Structure and Local Order of Lyotropic Cholesteric Calamitic Phases: The Effect of the Chiral Molecule,” Journal of Molecular Liquids 349 (2022): 118097, 10.1016/j.molliq.2021.118097.

[24] V. Tchakalova , C. L. P. Oliveira , and A. M. Figueiredo Neto , “New Lyotropic Complex Fluid Structured in Sheets of Ellipsoidal Micelles Solubilizing Fragrance Oils,” ACS Omega 8 (2023): 29568–29584, 10.1021/acsomega.3c03500. 37599987 PMC10433498

[25] M. Deutsch , “Orientational Order Determination in Liquid‐Crystals by X‐Ray‐Diffraction,” Physical Review A 44, no. 12 (1991): 8264–8270, 10.1103/PhysRevA.44.8264. 9905980

[26] D. Istrate , “Heat Induced Denaturation of Fibrous Hard Alpha‐Keratins and Their Reaction With Various Chemical Reagents” (PhD thesis, RWTH Aachen, 2011).

[27] C. R. R. C. Lima , R. A. A. Couto , T. B. Freire , et al., “Heat‐Damaged Evaluation of Virgin Hair,” Journal of Cosmetic Dermatology 18, no. 6 (2019): 1885–1892, 10.1111/jocd.12892. 30861299

[28] M. Brebu and I. Spiridon , “Thermal Degradation of Keratin Waste,” Journal of Analytical and Applied Pyrolysis 91, no. 2 (2011): 288–295, 10.1016/j.jaap.2011.03.003.

[29] F. J. Wortmann , G. Wortmann , J. Marsh , and K. Meinert , “Thermal Denaturation and Structural Changes of Alpha‐Helical Proteins in Keratins,” Journal of Structural Biology 177, no. 2 (2012): 553–560, 10.1016/j.jsb.2011.09.014. 22032853

[30] M. Baias , D. E. Demco , C. Popescu , et al., “Thermal Denaturation of Hydrated Wool Keratin by H‐1 Solid‐State NMR,” Journal of Physical Chemistry B 113, no. 7 (2009): 2184–2192, 10.1021/jp8094616. 19173568

[31] D. Istrate , C. Popescu , M. E. Rafik , and M. Moeller , “The Effect of pH on the Thermal Stability of Fibrous Hard Alpha‐Keratins,” Polymer Degradation and Stability 98, no. 2 (2013): 542–549, 10.1016/j.polymdegradstab.2012.12.001.

[32] L. Coderch , R. Di Lorenzo , M. Mussone , C. Alonso , and M. Martí , “The Role of Lipids in the Process of Hair Ageing,” Cosmetics 9, no. 6 (2022): 124, 10.3390/cosmetics9060124.

[33] L. Coderch , S. Mendez , C. Barba , R. Pons , M. Marti , and J. L. Parra , “Lamellar Rearrangement of Internal Lipids From Human Hair,” Chemistry and Physics of Lipids 155, no. 1 (2008): 1–6, 10.1016/j.chemphyslip.2008.05.175. 18619428

[34] M. Wade , I. Tucker , P. Cunningham , et al., “Investigating the Origins of Nanostructural Variations in Differential Ethnic Hair Types Using X‐Ray Scattering Techniques,” International Journal of Cosmetic Science 35, no. 5 (2013): 430–441, 10.1111/ics.12061. 23634942

[35] L. Bertrand , J. Doucet , A. Simionovici , G. Tsoucaris , and P. Walter , “Lead‐Revealed Lipid Organization in Human Hair,” Biochimica et Biophysica Acta‐General Subjects 1620, no. 1–3 (2003): 218–224, 10.1016/s0304-4165(02)00538-x. 12595092

[36] F. C. Yang , Y. Zhang , and M. C. Rheinstadter , “The Structure of People's Hair,” PeerJ 2 (2014): e619, 10.7717/peerj.619. 25332846 PMC4201279

[37] P. M. J. Barton , A Forensic Investigation of Single Human Hair Fibres Using FTIR‐ATR Spectroscopy and Chemometrics, Queensland University of Technology (Australy , 2011).

[38] Y. C. Zhang , R. J. Alsop , A. Soomro , F. C. Yang , and M. C. Rheinstadter , “Effect of Shampoo, Conditioner and Permanent Waving on the Molecular Structure of Human Hair,” PeerJ 3 (2015): e1296, 10.7717/peerj.1296. 26557428 PMC4636411

[39] Y. Yu , W. Yang , B. Wang , and M. A. Meyers , “Structure and Mechanical Behavior of Human Hair,” Materials Science & Engineering, C: Materials for Biological Applications 73 (2017): 152–163, 10.1016/j.msec.2016.12.008. 28183593

[40] P. M. J. Barton , “6th World Congress for Hair Research,” Experimental Dermatology 19, no. 6 (2010): 579, 10.1111/j.1600-0625.2010.01097.x.

[41] V. Stanic , J. Bettini , F. E. Montoro , A. Stein , and K. Evans‐Lutterodt , “Local Structure of Human Hair Spatially Resolved by Sub‐Micron X‐Ray Beam,” Scientific Reports 5 (2015): 17347, 10.1038/srep17347. 26617337 PMC4663634

[42] B. D. Cullity and S. R. Stock , Elements of X‐Ray Diffraction, 3rd ed. (Prentice Hall, 2001), xviii, 664s.

[43] E. Rafik , J. Doucet , and F. Briki , “The Intermediate Filament Architecture as Determined by X‐Ray Diffraction Modeling of Hard α‐Keratin,” Biophysical Journal 86, no. 6 (2004): 3893–3904, 10.1529/biophysj.103.034694. 15189886 PMC1304291

[44] V. F. Monteiro , A. P. Maciel , and E. Longo , “Thermal Analysis of Caucasian Human Hair,” Journal of Thermal Analysis and Calorimetry 79, no. 2 (2005): 289–293, 10.1007/s10973-005-0051-9.

[45] C. R. R. d. C. Lima , M. M. de Almeida , M. V. Robles Velasco , and J. d. R. Matos , “Thermoanalytical Characterization Study of Hair From Different Ethnicities,” Journal of Thermal Analysis and Calorimetry 123, no. 3 (2016): 2321–2328, 10.1007/s10973-015-5070-6.

[46] K. Sundaram , S. Gunasekaran , E. Sailatha , P. Marthandam , and P. Kuppuraj , “FTIR‐ATR Spectroscopic Technique on Human Single Intact Hair Fibre—A Case Study of Thyroid Patients,” International Journal of Advanced Scientific Technologies in Engineering and Management Sciences 2 (2016): 2454–2456.

[47] B. C. Smith , Infrared Spectral Interpretation: A Systematic Approach (CRC Press, 1998).

[48] C. Sandt and F. Borondics , “A New Typology of Human Hair Medullas Based on Lipid Composition Analysis by Synchrotron FTIR Microspectroscopy,” Analyst 146, no. 12 (2021): 3942–3954, 10.1039/d1an00695a. 33982696

[49] C. Bolduc and J. Shapiro , “Hair Care Products: Waving, Straightening, Conditioning, and Coloring,” Clinics in Dermatology 19, no. 4 (2001): 431–436, 10.1016/S0738-081X(01)00201-2. 11535384

[50] M. Al‐Kelani and N. Buthelezi , “Advancements in Medical Research: Exploring Fourier Transform Infrared (FTIR) Spectroscopy for Tissue, Cell, and Hair Sample Analysis,” Skin Research and Technology 30, no. 6 (2024): e13733, 10.1111/srt.13733. 38887131 PMC11182784

[51] G. Luengo , A. Fameau , F. Léonforte , and A. Greaves , “Surface Science of Cosmetic Substrates, Cleansing Actives and Formulations,” Advances in Colloid and Interface Science 290 (2021): 102383, 10.1016/j.cis.2021.102383. 33690071

[52] C. R. R. C. Lima , R. J. S. Lima , L. D. B. Machado , et al., “Human Hair: Subtle Change in the Thioester Groups Dynamics Observed by Combining Neutron Scattering, X‐Ray Diffraction and Thermal Analysis,” European Physical Journal Special Topics 229, no. 17–18 (2020): 2825–2832, 10.1140/epjst/e2020-900217-4.

[53] M. Korte , S. Akari , H. Kühn , N. Baghdadli , H. Möhwald , and G. Luengo , “Distribution and Localization of Hydrophobic and Ionic Chemical Groups at the Surface of Bleached Human Hair Fibers,” Langmuir 30, no. 41 (2014): 12124–12129, 10.1021/la500461y. 25203784

[54] C. Linch , S. Smith , and J. Prahlow , “Evaluation of the Human Hair Root for DNA Typing Subsequent to Microscopic Comparison,” Journal of Forensic Sciences 43, no. 2 (1998): 305–314. 9544538

[55] R. Bala , A. Sharma , and V. Sharma , “Animal Family Discrimination From Hair Using ATR‐FTIR and Machine Learning Methods for Applications in Illegal Wildlife Trafficking,” Science of Nature 111, no. 6 (2024): 59, 10.1007/s00114-024-01944-2. 39446166

[56] S. Nishikawa‐Torikai , M. Osawa , and S. Nishikawa , “Functional Characterization of Melanocyte Stem Cells in Hair Follicles,” Journal of Investigative Dermatology 131, no. 12 (2011): 2358–2367, 10.1038/jid.2011.195. 21753783

[57] S. Rao , D. Jain , J. Gaur , and R. Verma , “A Rapid and Non‐Destructive Identification of Animal Hairs Using ATR‐FTIR and Chemometrics: A Proof‐of‐Concept for Wildlife Forensic Applications,” Problems of Forensic Sciences 138 (2024): 137–152.

[58] E. O. Espinoza , B. W. Baker , T. D. Moores , and D. Voin , “Forensic Identification of Elephant and Giraffe Hair Artifacts Using HATR FTIR Spectroscopy and Discriminant Analysis,” Endangered Species Research 9 (2008): 239–246.

