# Supplementary material for: Thermal Induced Changes in Cuticle and Cortex to Chemically Treated Hair
Source: Biopolymers. 2025 Dec 13;117(1):e70071. doi: 10.1002/bip.70071 (PMC12701549; doi:10.1002/bip.70071)
Supplement: Supplementary file 1 — Data S1: Supporting Information. [file BIP-117-e70071-s001.pdf]

Supporting information for article:

Thermal Induced Changes in Virgin and Chemically Treated Hair

C. R. R. C. Lima, A.C. C. Bandeira , T. S. Martins, L. Otubo, C. L. P. Oliveira

Thermal induced changes in cuticle and cortex to chemically treated hair

C. R. R. C. Lima[1$], A.C. C. Bandeira[1$] , T. S. Martins[2], L. Otubo[3], C. L. P. Oliveira[1*]

[1]Institute of Physics, University of São Paulo, São Paulo 05508-090, SP, Brazil
[2]Institute of Environmental, Chemical and Pharmaceutical Sciences, Federal University of São Paulo, São Paulo 09913-030, SP, Brazil
[3]Institute for Energy and Nuclear Research, São Paulo 05508-000 SP, Brazil

[$] - first author
corresponding author: crislpo@if.usp.br

Supplementary Information

1- In-situ temperature-variation X-ray scattering measurements of the hair samples

As described in the main text – Line 140 – the virgin and chemically treated hair samples (VH, BH, SH and BSH) were placed in the sample holder and inserted in SAXS equipment. The apparatus used to mount the hair fibers in the experiment is in *Figure S1*. Around 30 fibers were mounted in the support.

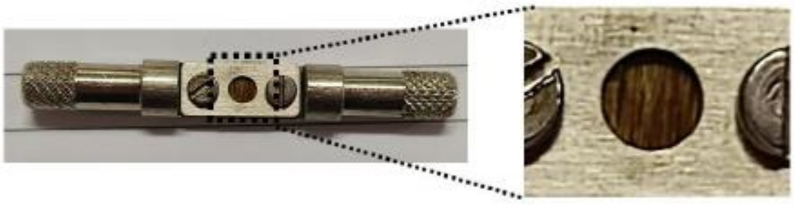

Figure S1 – Hair tresses mounting for chamber for a controlled heating using X-Ray scattering measurements.

For in-situ X-ray scattering measurements, a Hot Stage heating sample holder from MRI was used in the Xeuss2 chamber – described at Line 156 in the text of paper and showed here in Figure S2. This heating system includes the 'Hot Stage' and a 'Chiller,' which acts as a thermal reservoir to cool the heating control unit. X-ray measurements with heating were conducted at temperatures of 30, 50, 60, 70, 80, 90, 100, 110, 120, 130, 140, 150, 175, 200, 225, 230, 245, 250, 260, 275 and 300°C.

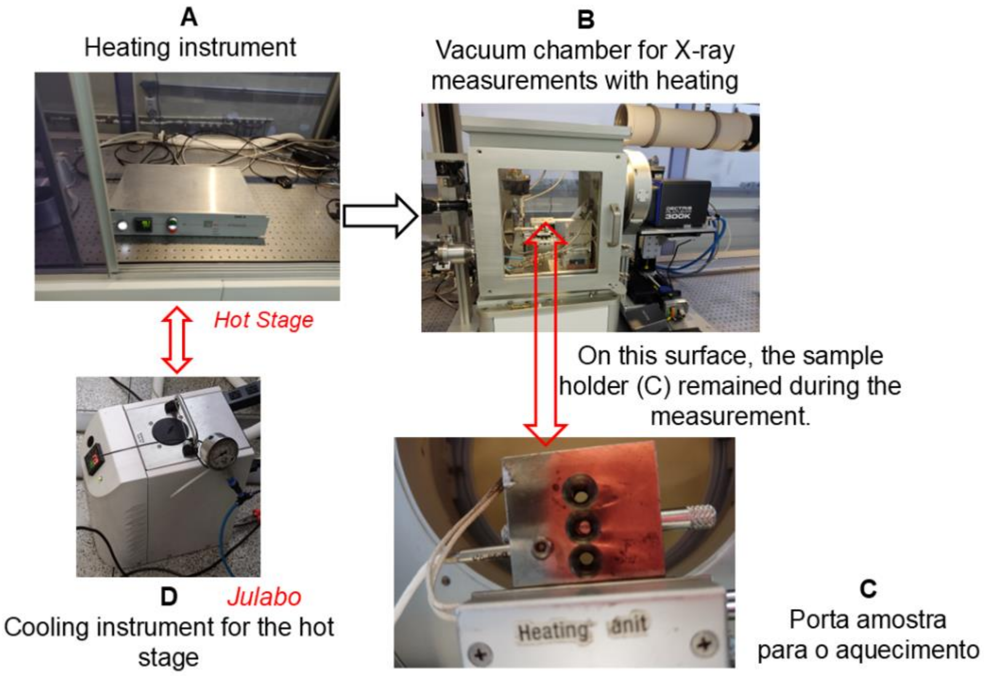

Figure S2 – Hot Stage heating sample holder from MRI used in the Xeuss2 chamber for a controlled heating of the hair sample.

2- In-situ measurements X-ray data for hair tresses to subjected to temperature-controlled program

The angular sectors of the 2D images were taken and combined in singe 1D curves as presented in Figure 1 (a) showed in the text of paper. Figure 1 (b) shows the scattering across the fiber (equatorial direction) and Figure 1 (c) along the fiber (meridional direction) for virgin hair samples (VH).

Figure S3 below presents USAXS, SAXS and WAXS scattering of the evolution of behavior of the chemically treated caucasian hair tresses (BH, SH and BSH) under heating for 20 minutes at 30, 50, 60, 70, 80, 90, 100, 110, 120, 130, 140, 150, 175, 200, 225, 230, 245, 250, 260, 275 and 300°C in-situ X-ray measurements.

2.1- Virgin hair (BH)

The angular sectors of the 2D images were taken and combined in singe 1D curves as presented in Figure S3 (d). Figure S3 (e) shows the scattering across the fiber (equatorial direction) and Figure S3 (f) along the fiber (meridional direction) for virgin hair samples.

2.2- Bleached hair (BH)

The angular sectors of the 2D images were taken and combined in singe 1D curves as presented in Figure S3 (d). Figure S3 (e) shows the scattering across the fiber (equatorial direction) and Figure S3 (f) along the fiber (meridional direction) for bleached hair samples.

2.3- Straightened hair (SH)

The angular sectors of the 2D images were taken and combined in singe 1D curves as presented in Figure S3 (g). Figure S3 (h) shows the scattering across the fiber (equatorial direction) and Figure S3 (i) along the fiber (meridional direction) for straightened hair samples.

2.4- Bleached and Straightening hair (BSH)

The angular sectors of the 2D images were taken and combined in singe 1D curves as presented in Figure S3 (j). Figure S3 (k) shows the scattering across the fiber (equatorial direction) and Figure S3 (l) along the fiber (meridional direction) for bleached and straightening hair samples.

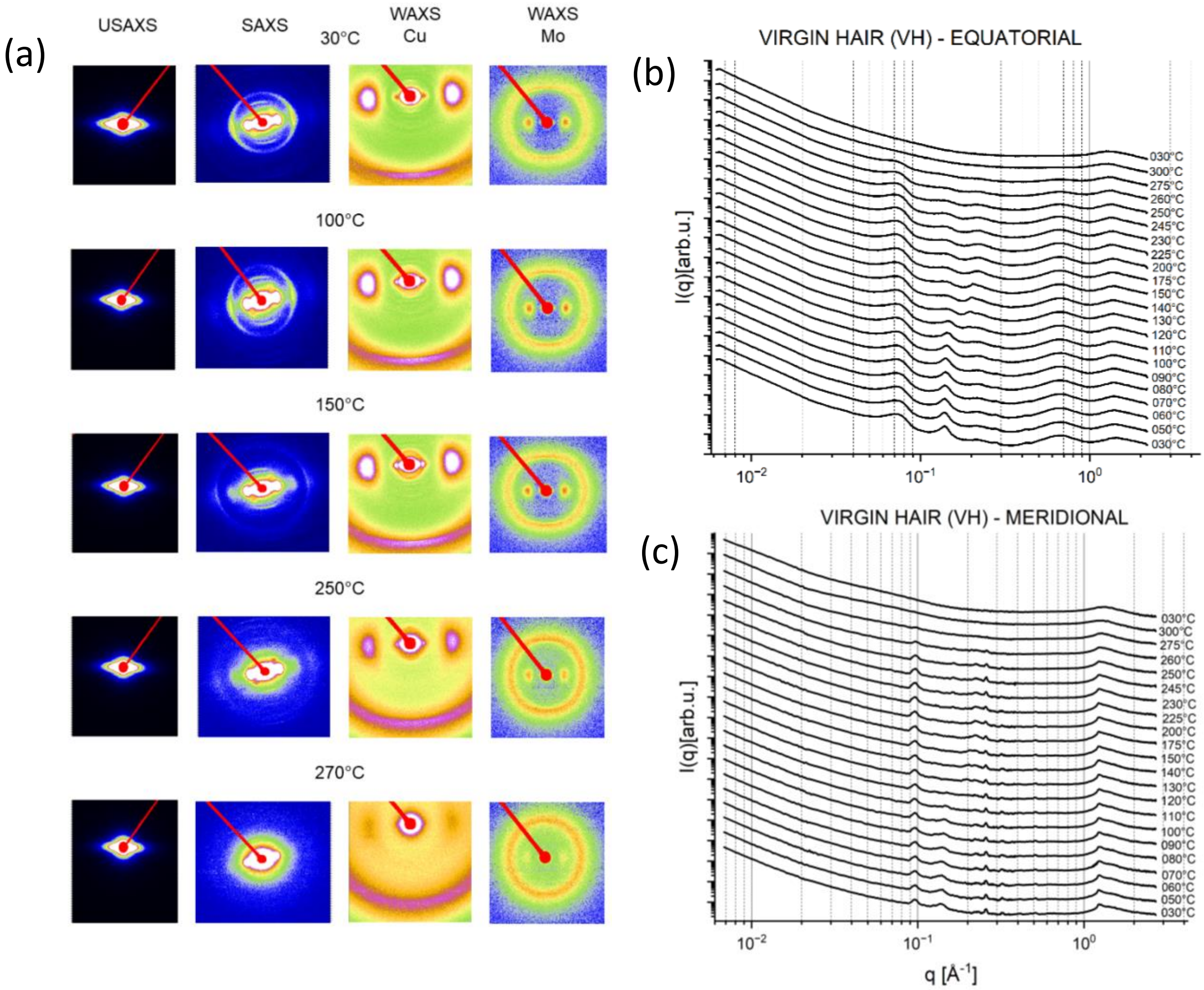

Figure S3 - (a) 2D images of USAXS, SAXS, WAXS – Cu and WAXS – Mo of virgin hair at temperatures: 30°C, 100°C, 150°C, 250°C and 270°C. USAXS, SAXS and WAXS curves in the equatorial **(b)** and meridional **(c)** cuts (section) of bleached hair at temperatures: 30, 50, 60, 70, 80, 90, 100, 110, 120, 130, 140, 150, 175, 200, 225, 230, 245, 250, 260, 275 and 300°C.

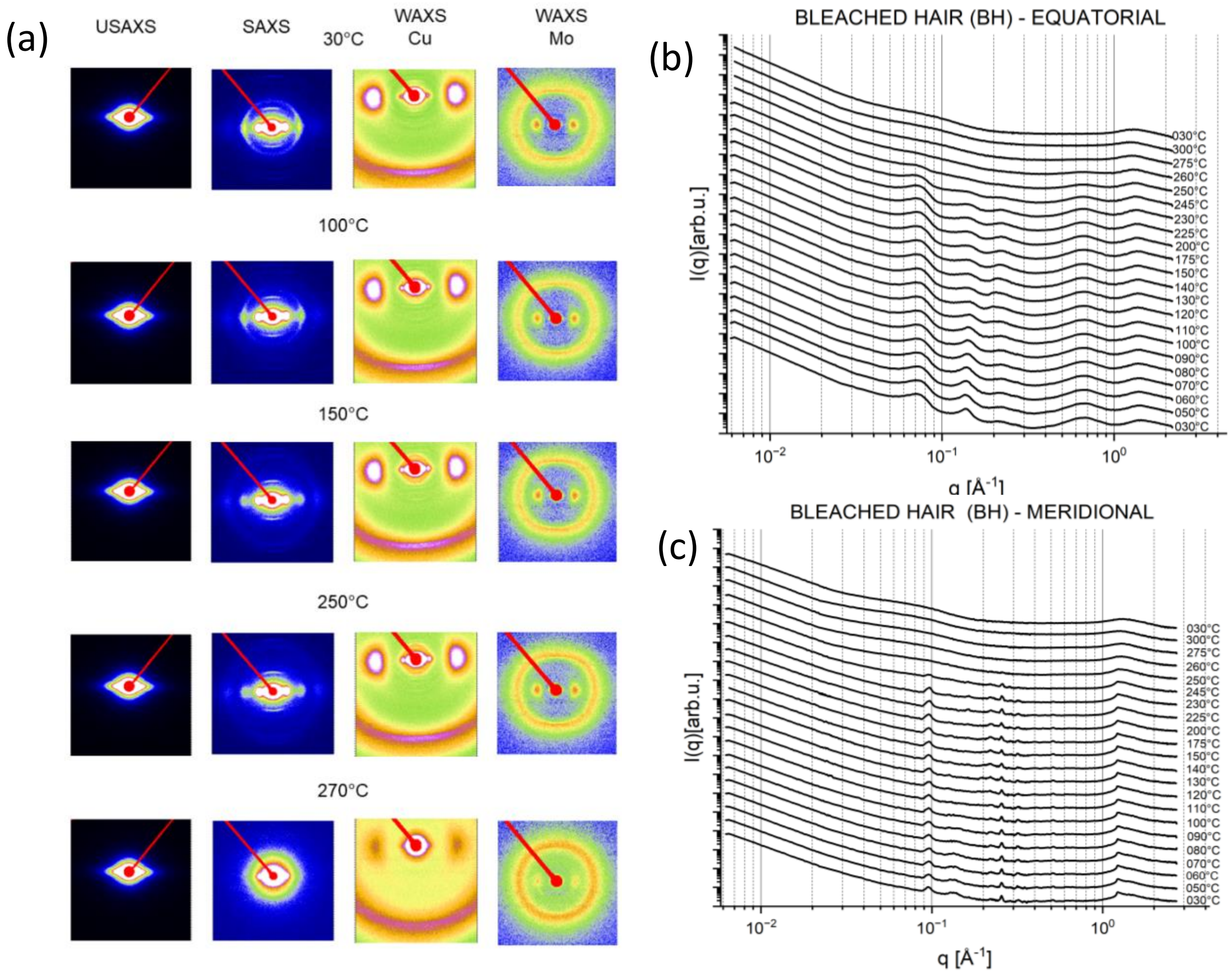

Figure S4 - (a) 2D images of USAXS, SAXS, WAXS – Cu and WAXS – Mo of bleached hair at temperatures: 30°C, 100°C, 150°C, 250°C and 270°C. USAXS, SAXS and WAXS curves in the equatorial **(b)** and meridional **(c)** cuts (section) of bleached hair at temperatures: 30, 50, 60, 70, 80, 90, 100, 110, 120, 130, 140, 150, 175, 200, 225, 230, 245, 250, 260, 275 and 300°C.

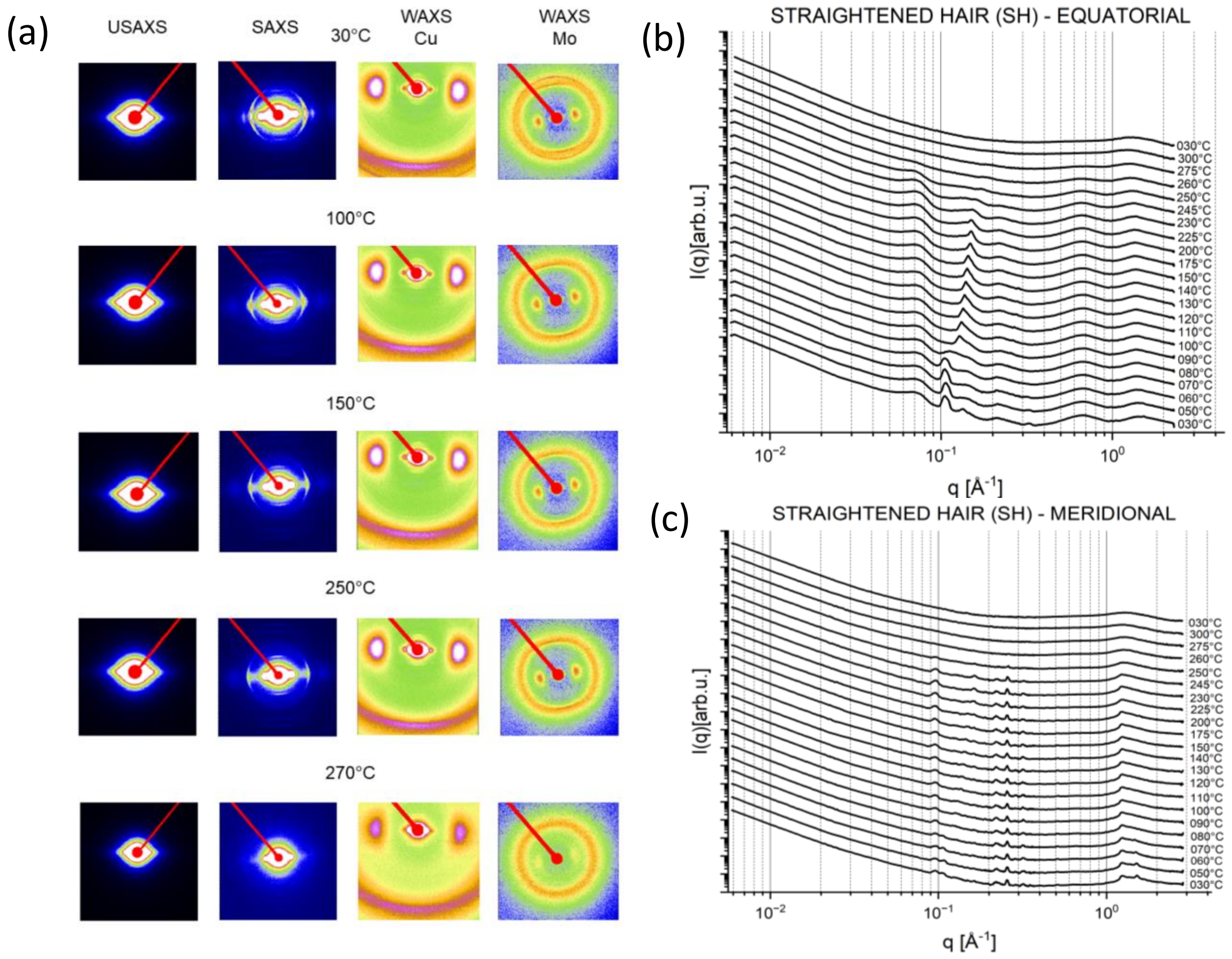

Figure S5 - **(a)** 2D images of USAXS, SAXS, WAXS – Cu and WAXS – Mo of straightened hair at temperatures: 30°C, 100°C, 150°C, 250°C and 270°C. USAXS, SAXS and WAXS curves in the equatorial **(b)** and meridional **(c)** cuts (section) of straightened hair at temperatures: 30, 50, 60, 70, 80, 90, 100, 110, 120, 130, 140, 150, 175, 200, 225, 230, 245, 250, 260, 275 and 300°C.

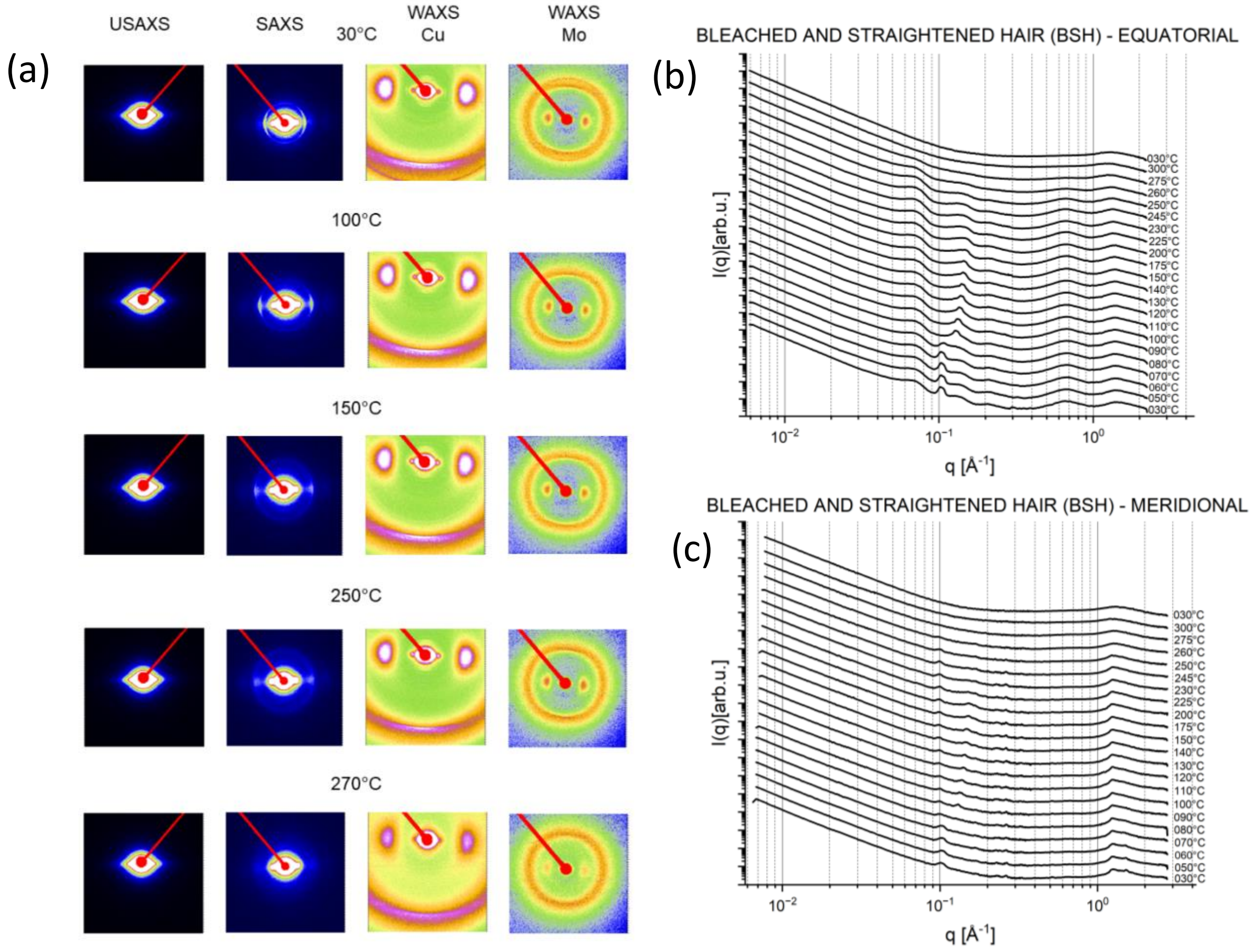

Figure S6 - (a) 2D images of USAXS, SAXS, WAXS – Cu and WAXS – Mo of bleached and straightened hair at temperatures: 30°C, 100°C, 150°C, 250°C and 270°C. USAXS, SAXS and WAXS curves in the equatorial **(b)** and meridional **(c)** cuts (section) of bleached and straightened hair at temperatures: 30, 50, 60, 70, 80, 90, 100, 110, 120, 130, 140, 150, 175, 200, 225, 230, 245, 250, 260, 275 and 300°C.

3- Scanning electron microscopy images (SEM)

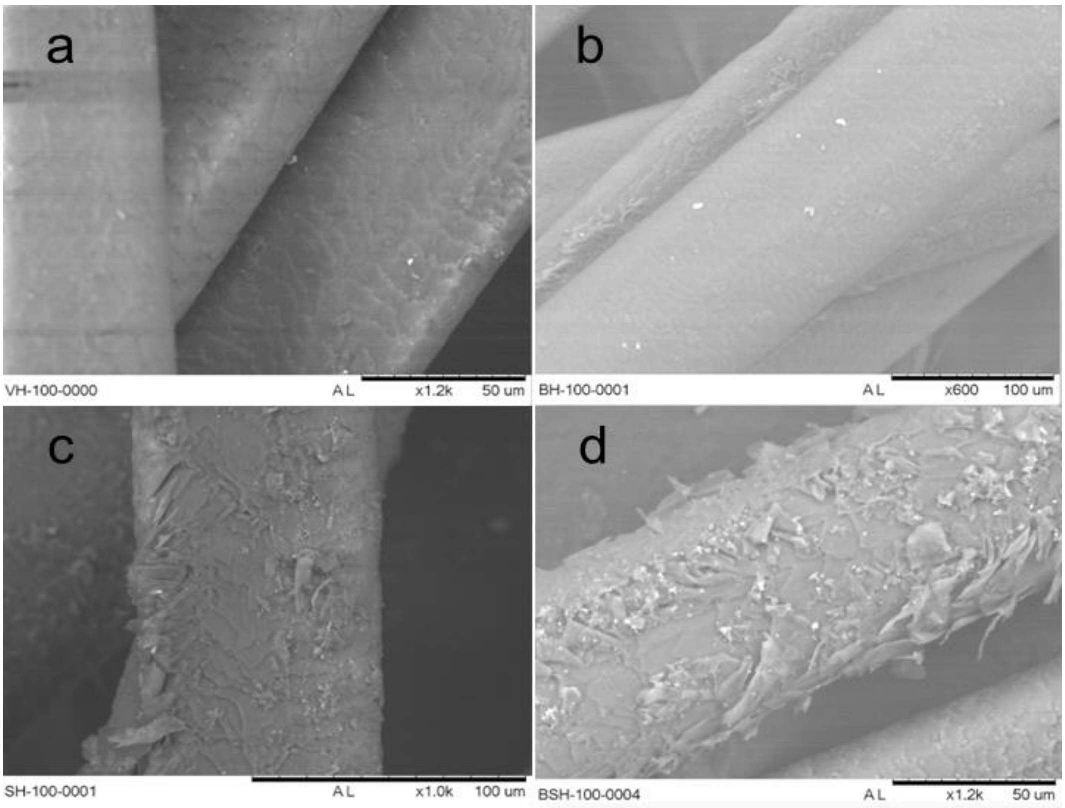

Figure S7 - SEM images of virgin (VH - a), bleached (BH - b), straightening (SH - c), bleached and straightening (BSH - d) at 100°C.

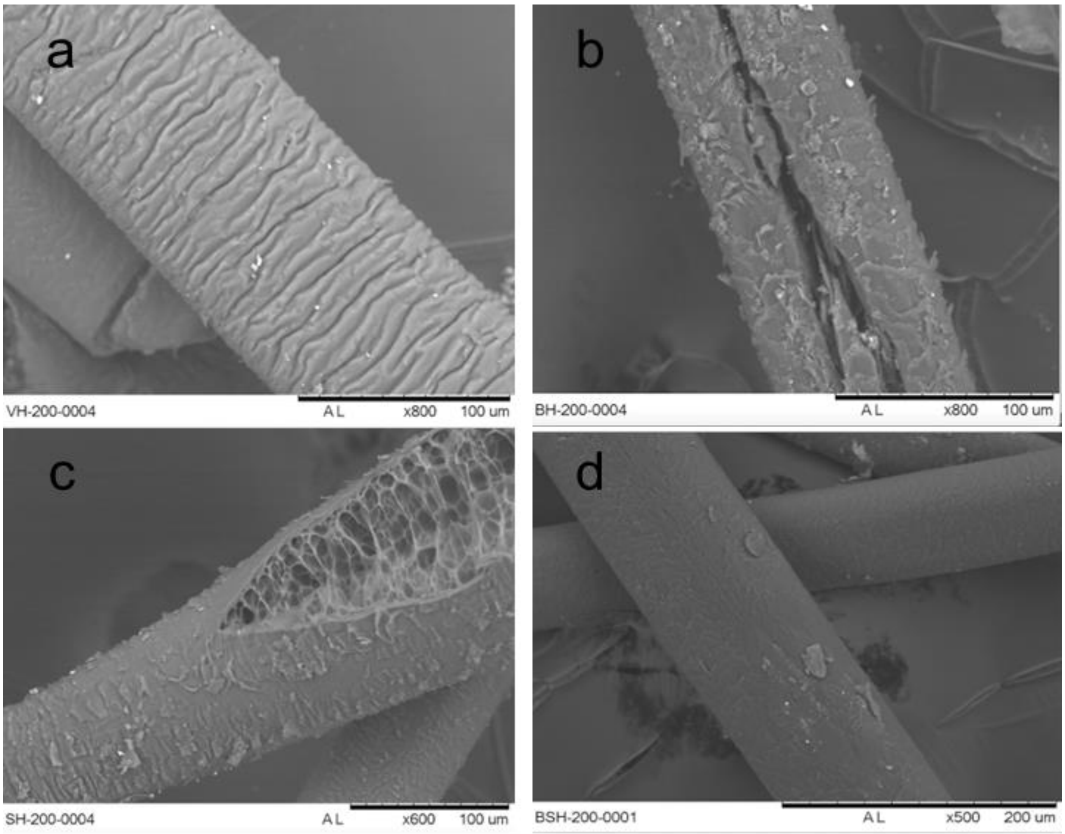

Figure S8 - SEM images of virgin (VH - a), bleached (BH - b), straightening (SH - c), bleached and straightening (BSH - d) at 200°C.

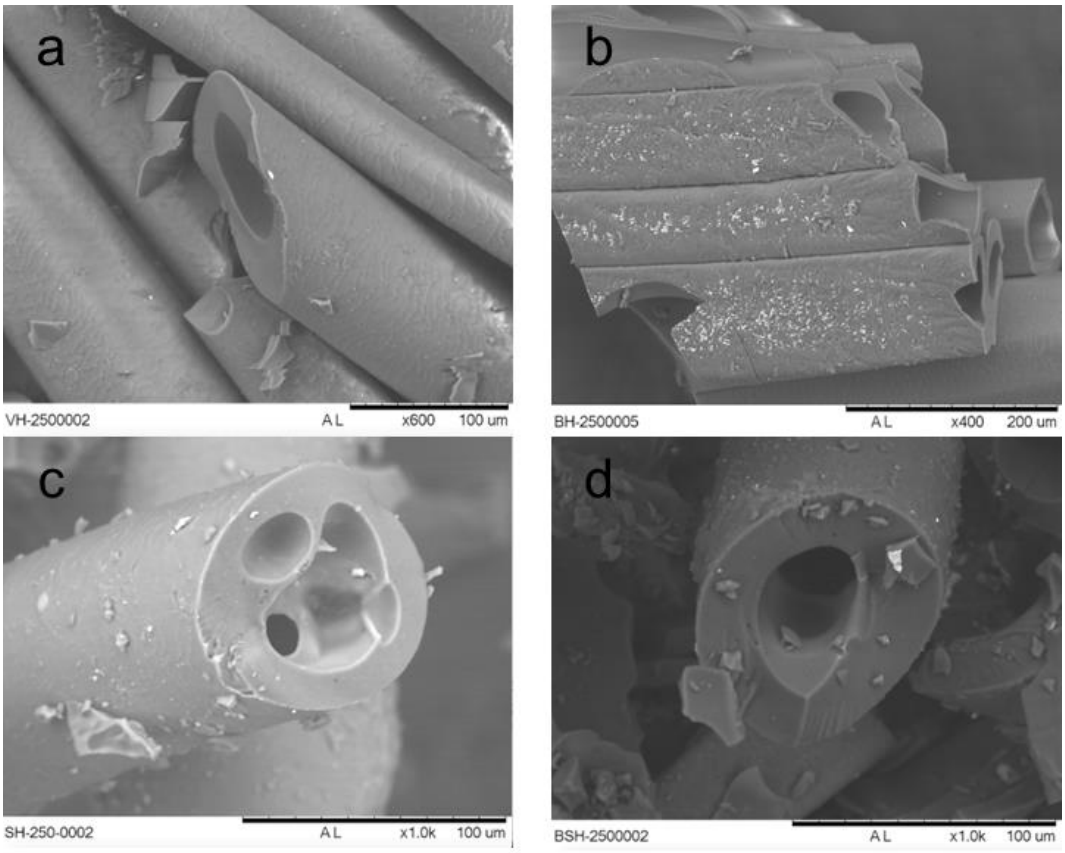

Figure S9 - SEM images of virgin (VH - a), bleached (BH - b), straightening (SH - c), bleached and straightening (BSH - d) at 250°C.

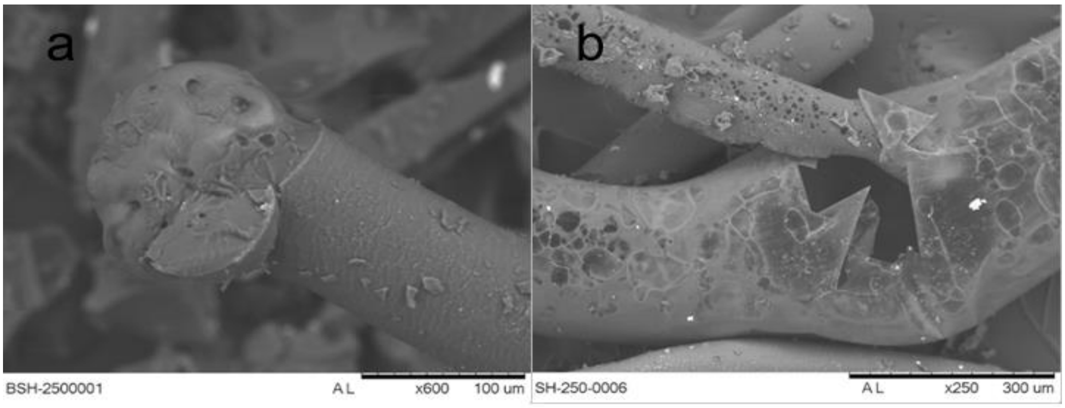

Figure S10 - SEM images of bleached and straightening (BSH - a) and straightening (SH - b) at 250°C.

4- Definition of angular slices in the X-Ray scattering images

In figures S11-S26 are shown the modeling results for the radial integrations. The fits for the oriented peaks were made using equation 17.

VH-SAXS-RADIAL

(a) (b)

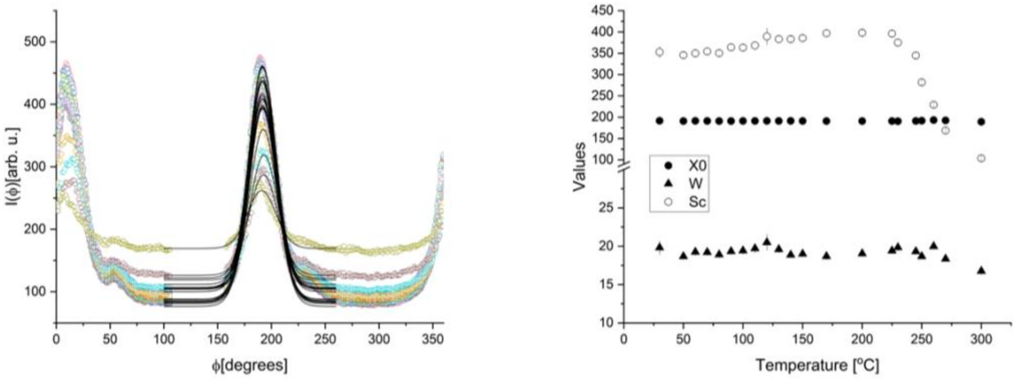

Figure S11 – Radial plots and modeling results for virgin hair (VH) for SAXS data. (a) radial plots (symbols) for the investigated temperatures and modeling results (solid lines) for the images of oriented peaks. (b) modeling results obtained from the fits, indicating that scattering data has an intrinsic orientation.

VH-USAXS-RADIAL

(a) (b)

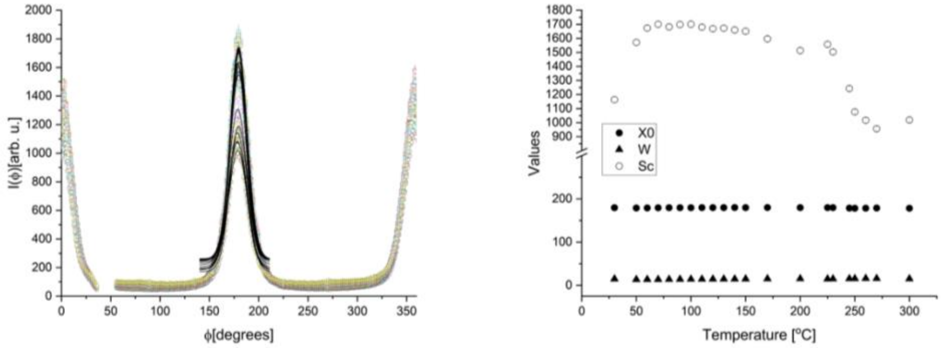

Figure S12 – Radial plots and modeling results for virgin hair (VH) for USAXS data. (a) radial plots (symbols) for the investigated temperatures and modeling results (solid lines) for the images of oriented peaks. (b) modeling results obtained from the fits, indicating that scattering data has an intrinsic orientation.

VH-WAXS-Cu-RADIAL

(a) (b)

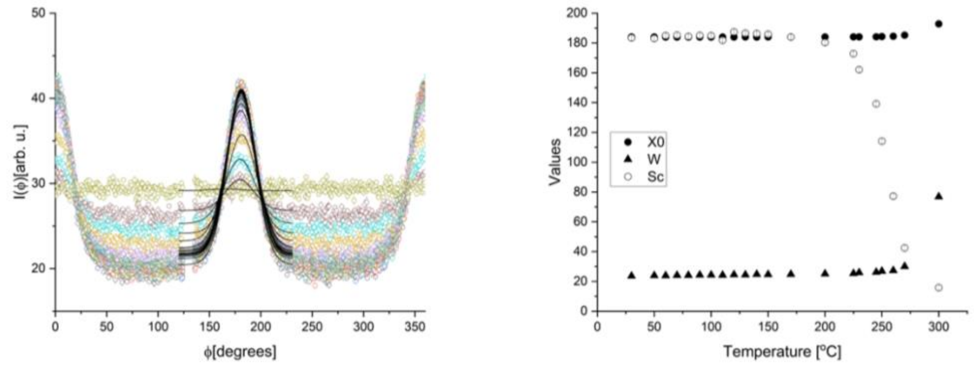

Figure S13 – Radial plots and modeling results for virgin hair (VH) for WAXS-Cu data. (a) radial plots (symbols) for the investigated temperatures and modeling results (solid lines) for the images of oriented peaks. (b) modeling results obtained from the fits, indicating that scattering data has an intrinsic orientation.

VH-WAXS-Mo-RADIAL

(a) (b)

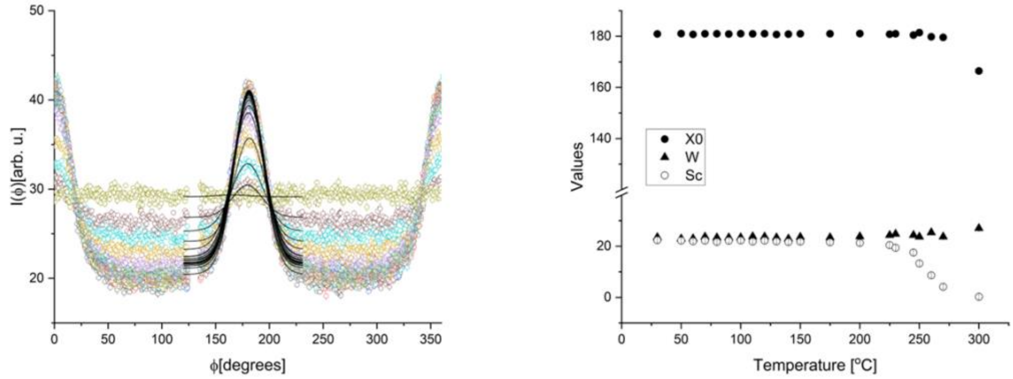

Figure S14 – Radial plots and modeling results for virgin hair (VH) for WAXS-Mo data. (a) radial plots (symbols) for the investigated temperatures and modeling results (solid lines) for the images of oriented peaks. (b) modeling results obtained from the fits, indicating that scattering data has an intrinsic orientation.

SH – SAXS-RADIAL

(a) (b)

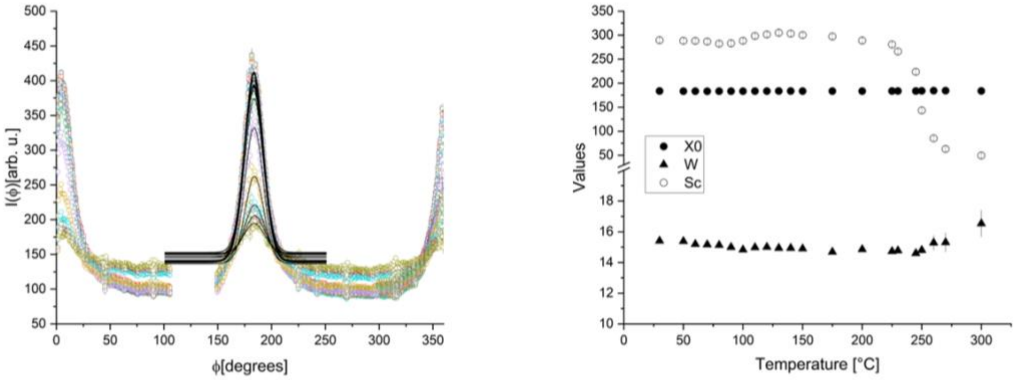

Figure S15 – Radial plots and modeling results for straightened hair (SH) for SAXS data. (a) radial plots (symbols) for the investigated temperatures and modeling results (solid lines) for the images of oriented peaks. (b) modeling results obtained from the fits, indicating that scattering data has an intrinsic orientation.

SH-USAXS-RADIAL

(a) (b)

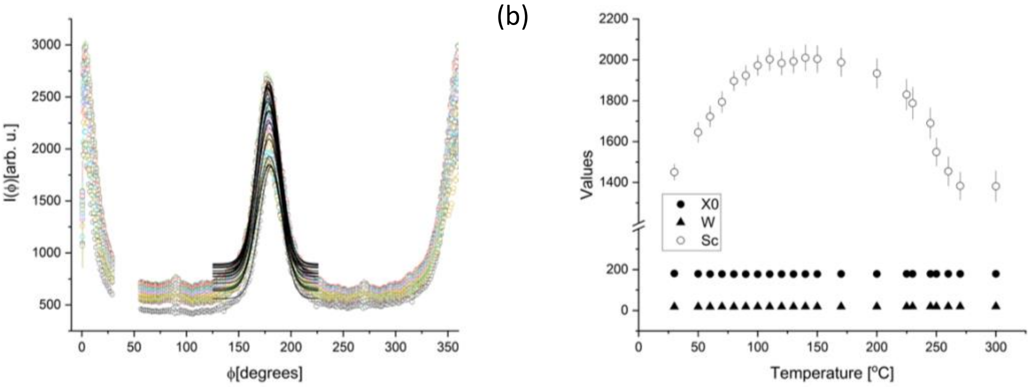

Figure S16 – Radial plots and modeling results for straightened hair (SH) for USAXS data. (a) radial plots (symbols) for the investigated temperatures and modeling results (solid lines) for the images of oriented peaks. (b) modeling results obtained from the fits, indicating that scattering data has an intrinsic orientation.

SH-WAXS-Cu-RADIAL

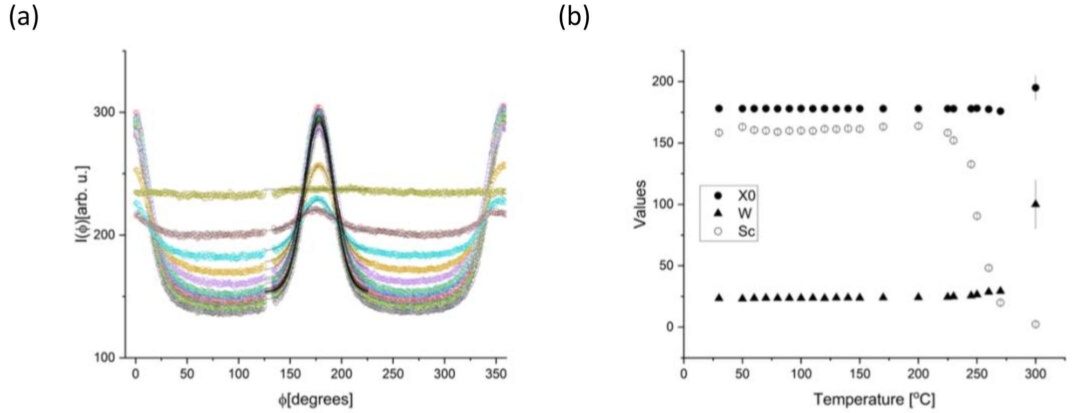

Figure S17 – Radial plots and modeling results for straightened hair (SH) for WAXS-Cu data. (a) radial plots (symbols) for the investigated temperatures and modeling results (solid lines) for the images of oriented peaks. (b) modeling results obtained from the fits, indicating that scattering data has an intrinsic orientation.

SH-WAXS-Mo-RADIAL

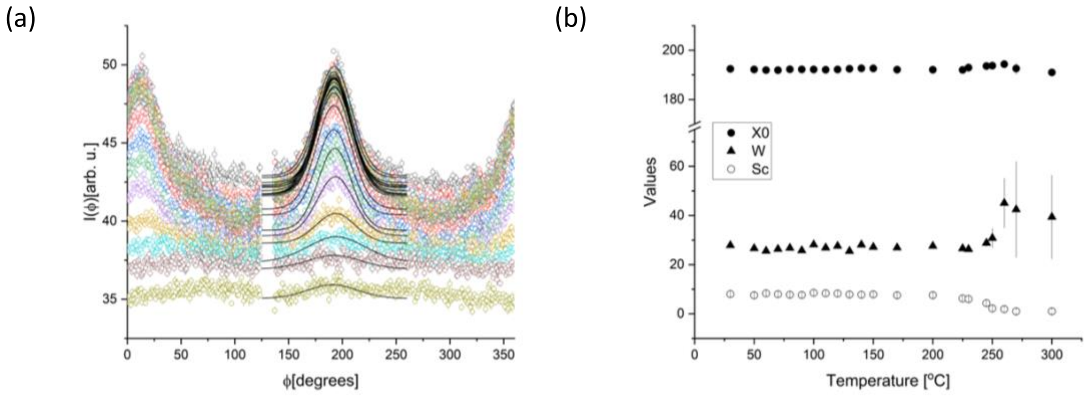

Figure S18 – Radial plots and modeling results for straightened hair (SH) for WAXS-Mo data. (a) radial plots (symbols) for the investigated temperatures and modeling results (solid lines) for the images of oriented peaks. (b) modeling results obtained from the fits, indicating that scattering data has an intrinsic orientation.

BSH-SAXS-RADIAL

(a) (b)

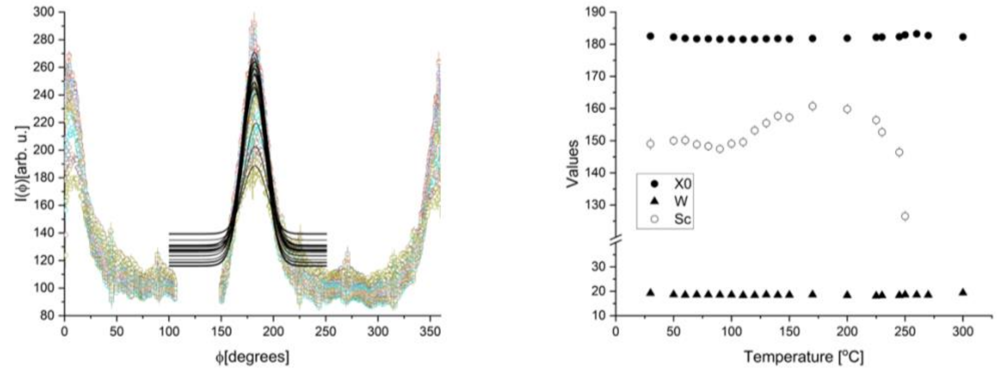

Figure S19– Radial plots and modeling results for bleached and straightened hair (BSH) for SAXS data. (a) radial plots (symbols) for the investigated temperatures and modeling results (solid lines) for the images of oriented peaks. (b) modeling results obtained from the fits, indicating that scattering data has an intrinsic orientation.

BSH-USAXS-RADIAL

(a) (b)

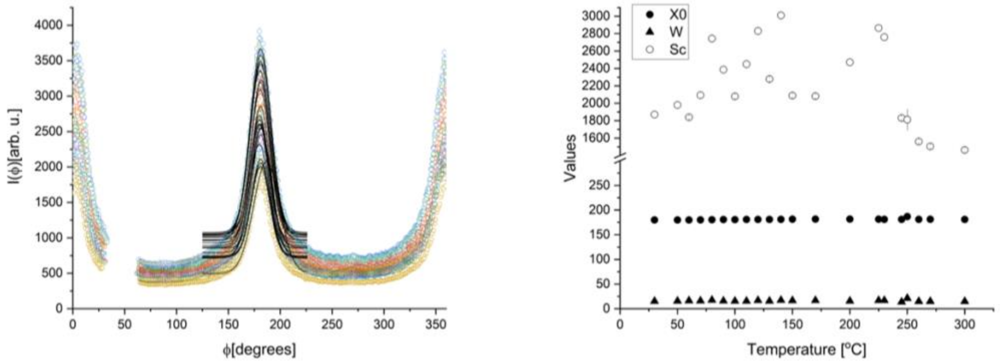

Figure S20 – Radial plots and modeling results for bleached and straightened hair (BSH) for USAXS data. (a) radial plots (symbols) for the investigated temperatures and modeling results (solid lines) for the images of oriented peaks. (b) modeling results obtained from the fits, indicating that scattering data has an intrinsic orientation.

BSH-WAXS-Cu-RADIAL

(a) (b)

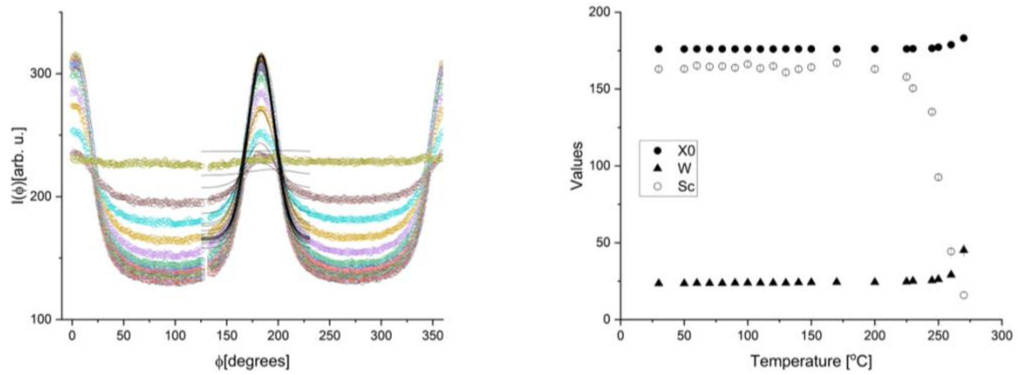

Figure S21 – Radial plots and modeling results for bleached and straightened hair (BSH) for WAXS-Cu data. (a) radial plots (symbols) for the investigated temperatures and modeling results (solid lines) for the images of oriented peaks. (b) modeling results obtained from the fits, indicating that scattering data has an intrinsic orientation.

BSH-WAXS-Mo-RADIAL

(a) (b)

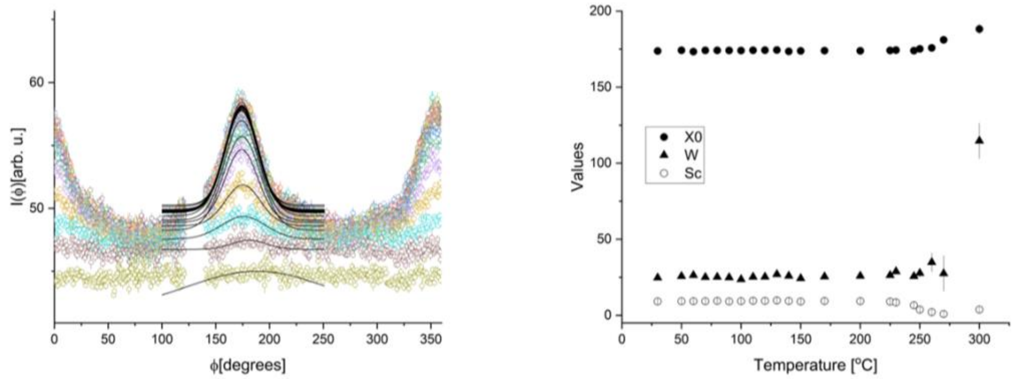

Figure S22 – Radial plots and modeling results for bleached and straightened hair (BSH) for SAXS data. (a) radial plots (symbols) for the investigated temperatures and modeling results (solid lines) for the images of oriented peaks. (b) modeling results obtained from the fits, indicating that scattering data has an intrinsic orientation.

BH-SAXS-RADIAL

(a) (b)

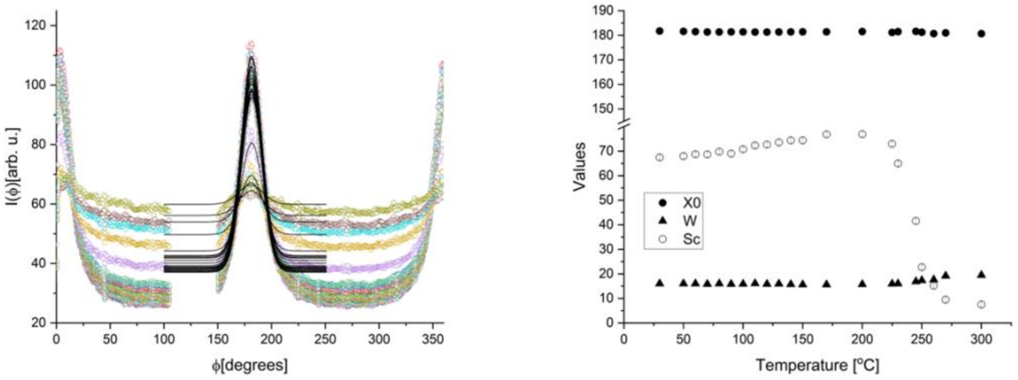

Figure S23 – Radial plots and modeling results for straightened hair (SH) for SAXS data. (a) radial plots (symbols) for the investigated temperatures and modeling results (solid lines) for the images of oriented peaks. (b) modeling results obtained from the fits, indicating that scattering data has an intrinsic orientation.

BH-USAXS-RADIAL

(a) (b)

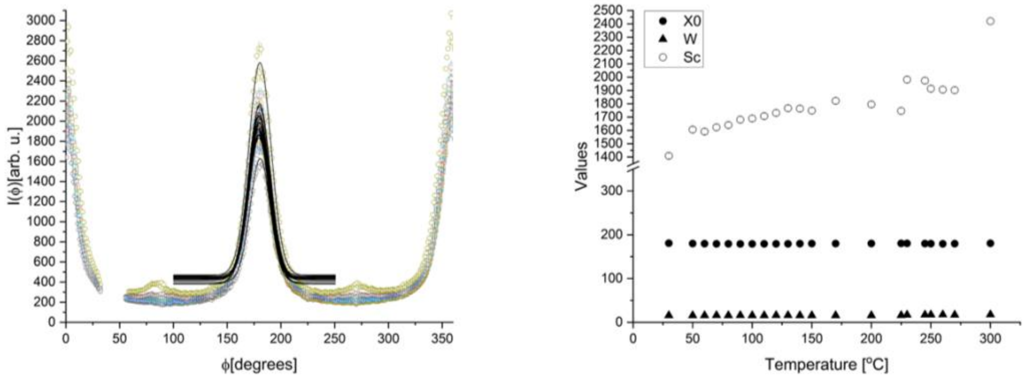

Figure S24 – Radial plots and modeling results for bleached hair (BH) for SAXS data. (a) radial plots (symbols) for the investigated temperatures and modeling results (solid lines) for the images of oriented peaks. (b) modeling results obtained from the fits, indicating that scattering data has an intrinsic orientation.

BH-WAXS-Cu-RADIAL

(a) (b)

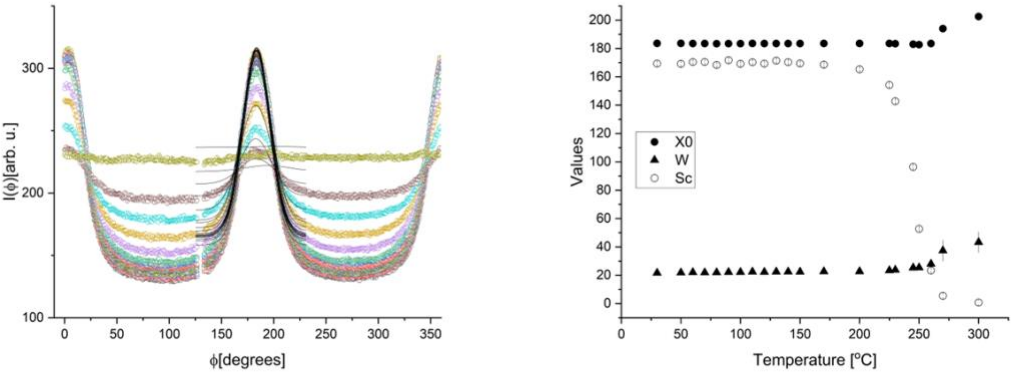

Figure S25 – Radial plots and modeling results for bleached hair (BH) for SAXS data. (a) radial plots (symbols) for the investigated temperatures and modeling results (solid lines) for the images of oriented peaks. (b) modeling results obtained from the fits, indicating that scattering data has an intrinsic orientation.

BH-WAXS-Mo-RADIAL

(a) (b)

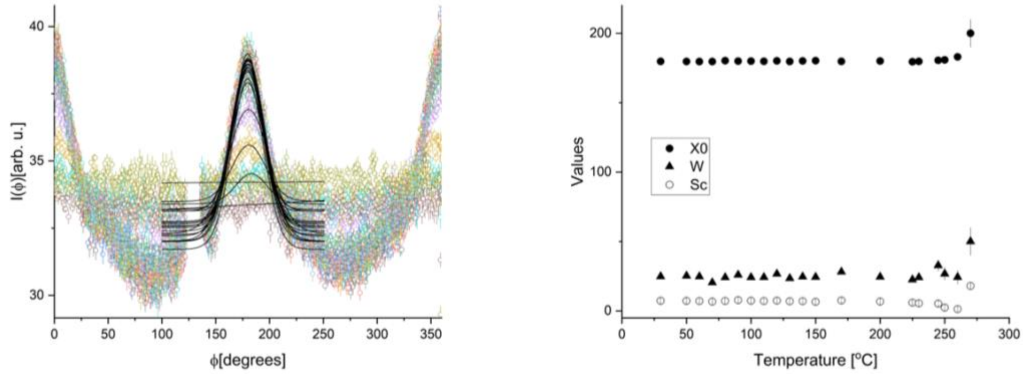

Figure S26 – Radial plots and modeling results for bleached hair (BH) for SAXS data. (a) radial plots (symbols) for the investigated temperatures and modeling results (solid lines) for the images of oriented peaks. (b) modeling results obtained from the fits, indicating that scattering data has an intrinsic orientation.

5- Modeling results for X-Ray scattering data

The USAXS, SAXS and WAXS data were modeled using equations (2) and (6). The results are shown below for each hair type.

a. Vigin hair – Equatorial cuts

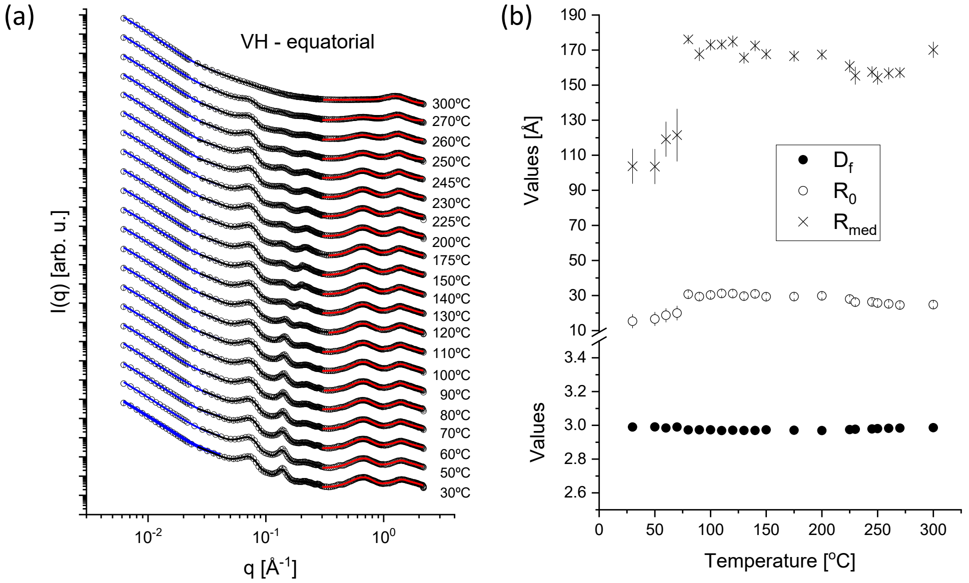

Figure S27 – Results for the modeling of X-ray scattering data. (a) X-ray scattering curves for each temperature (symbols) and theoretical curves (solid lines). Blue curves: USAXS fit, black curves: SAXS fit, red curves: WAXS fits. (b) results for the USAXS fit. For details, see article text.

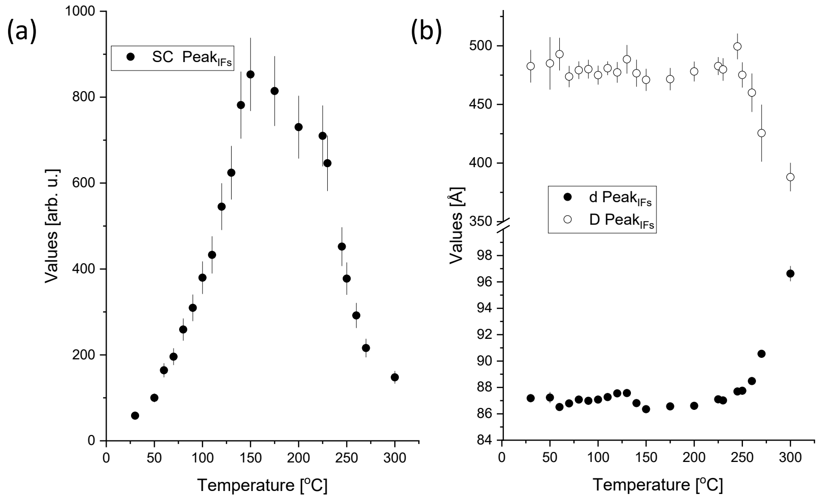

Figure S28 – Results for the modeling of X-ray scattering data. (a) scale factor for peak contribution of IFs region. (b) periodicity and domain size for IFs region. For details, see article text.

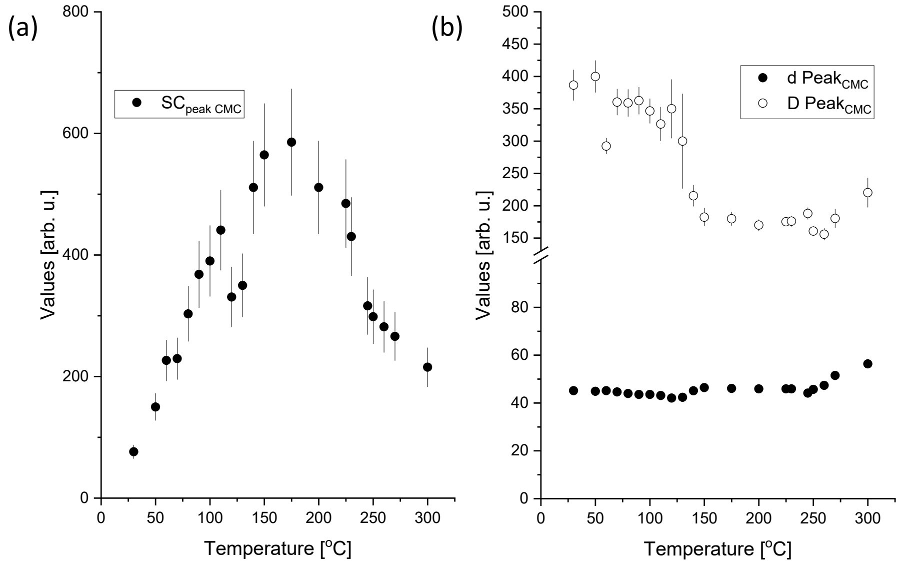

Figure S29 – Results for the modeling of X-ray scattering data. (a) scale factor for peak contribution of CMC region. (b) periodicity and domain size for CMC region. For details, see article text.

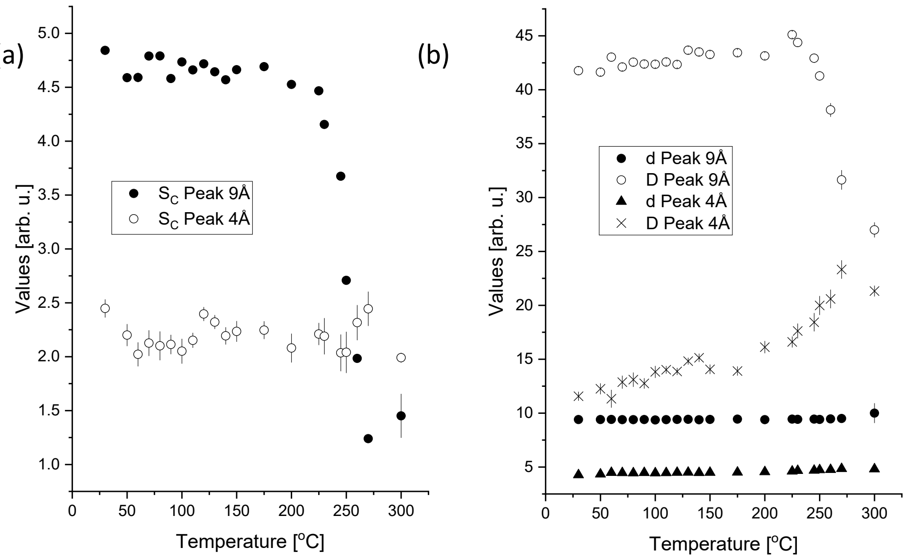

Figure S30 – Results for the modeling of X-ray scattering data. (a) scale factor for the peaks of 9Å and 4Å at WAXS region. (b) periodicity and domain size for the peaks of 9Å and 4Å at WAXS region. For details, see article text.

b. Vigin hair – Meridional cuts

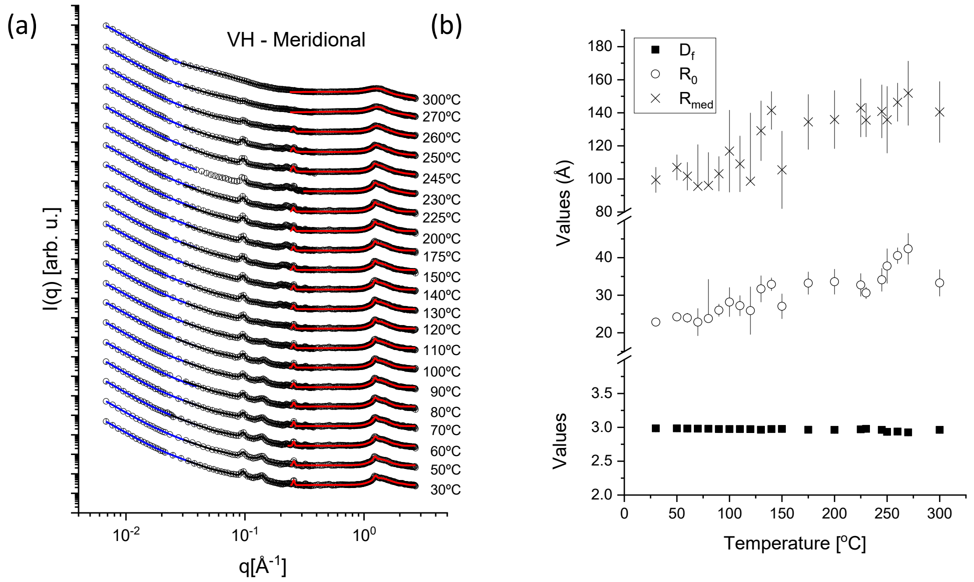

Figure S31 – Results for the modeling of X-ray scattering data. (a) X-ray scattering curves for each temperature (symbols) and theoretical curves (solid lines). Blue curves: USAXS fit, black curves: SAXS fit, red curves: WAXS fits. (b) results for the USAXS fit. For details, see article text.

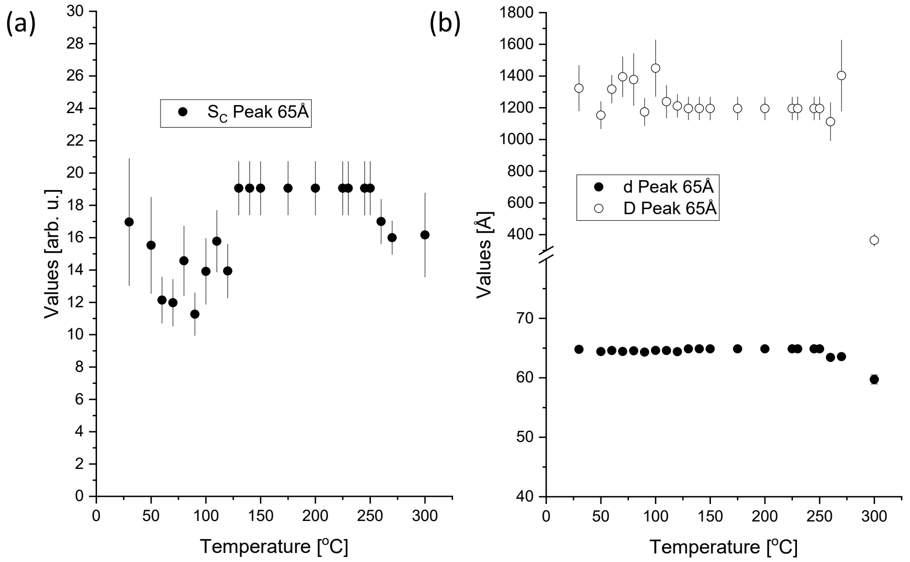

Figure S32 – Results for the modeling of X-ray scattering data. (a) scale factor for the peak of 65Å at SAXS region. (b) periodicity and domain size for the peak of 65Å. For details, see article text.

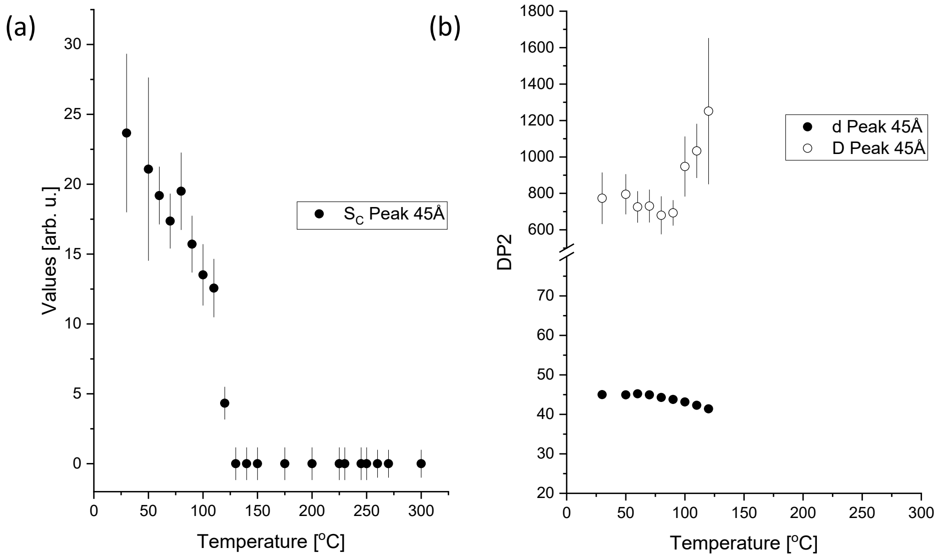

Figure S33 – Results for the modeling of X-ray scattering data. (a) scale factor for the peak of 45Å at SAXS region. (b) periodicity and domain size for the peak of 45Å. For details, see article text.

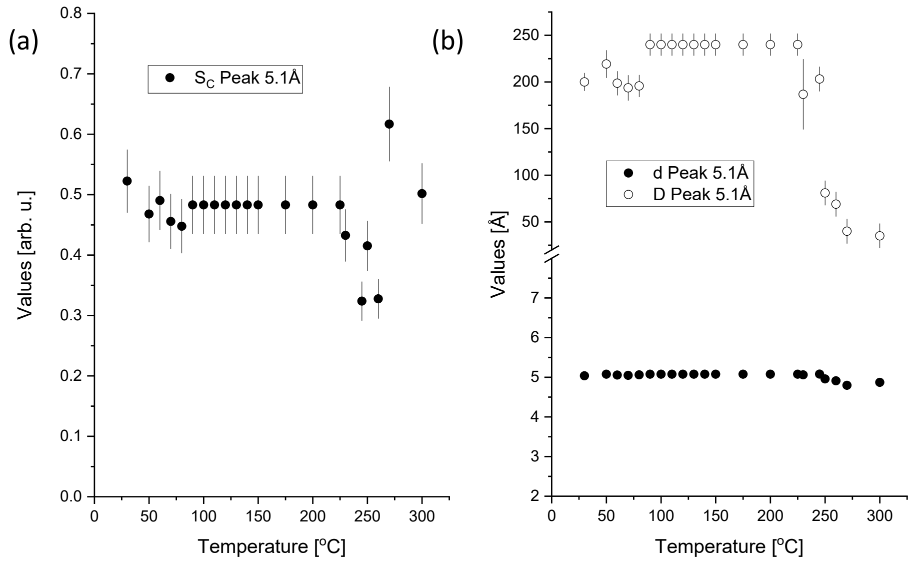

Figure S34 – Results for the modeling of X-ray scattering data. (a) scale factor for the peaks of 5.1Å at WAXS region. (b) periodicity and domain size for the peaks of 5.1Å WAXS region. For details, see article text.

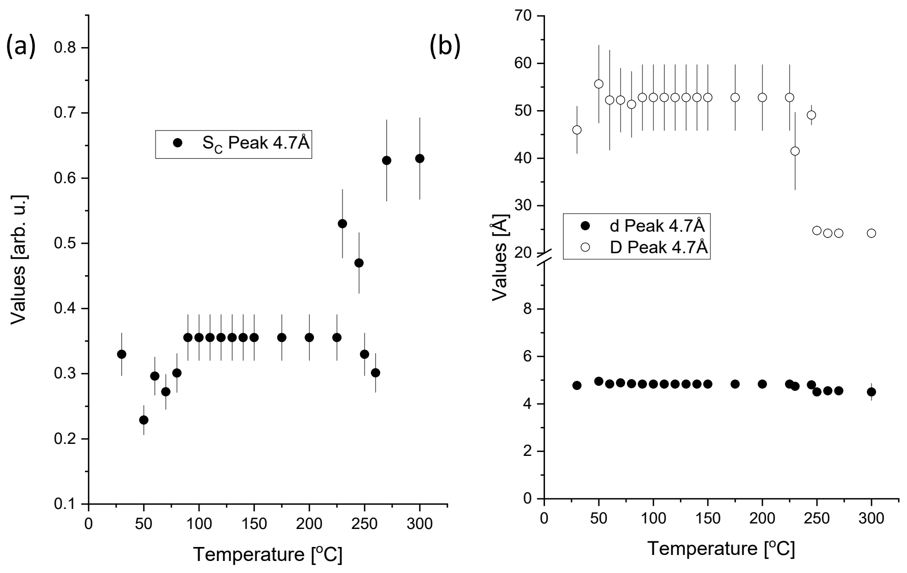

Figure S35 – Results for the modeling of X-ray scattering data. (a) scale factor for the peaks of 4.7Å at WAXS region. (b) periodicity and domain size for the peaks of 4.7Å WAXS region. For details, see article text.

a. Bleached hair – Equatorial cuts

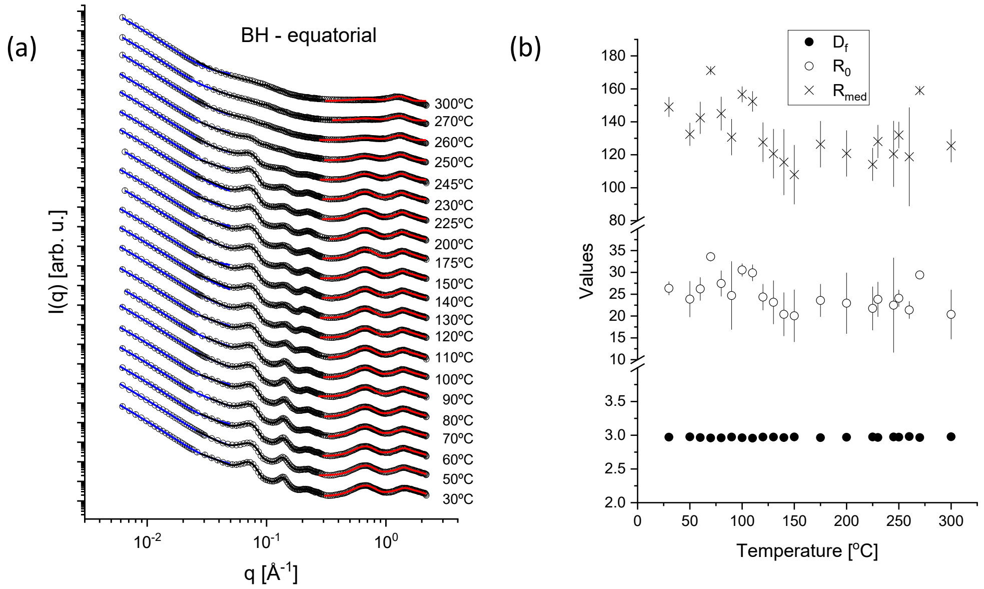

Figure S36 – Results for the modeling of X-ray scattering data. (a) X-ray scattering curves for each temperature (symbols) and theoretical curves (solid lines). Blue curves: USAXS fit, black curves: SAXS fit, red curves: WAXS fits. (b) results for the USAXS fit. For details, see article text.

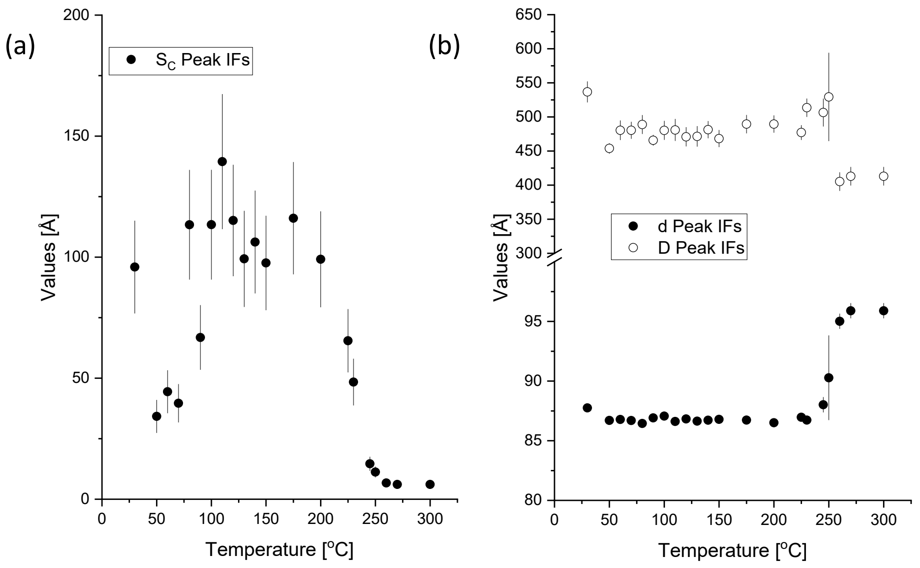

Figure S37– Results for the modeling of X-ray scattering data. (a) scale factor for peak contribution of IFs region. (b) periodicity and domain size for IFs region. For details, see article text.

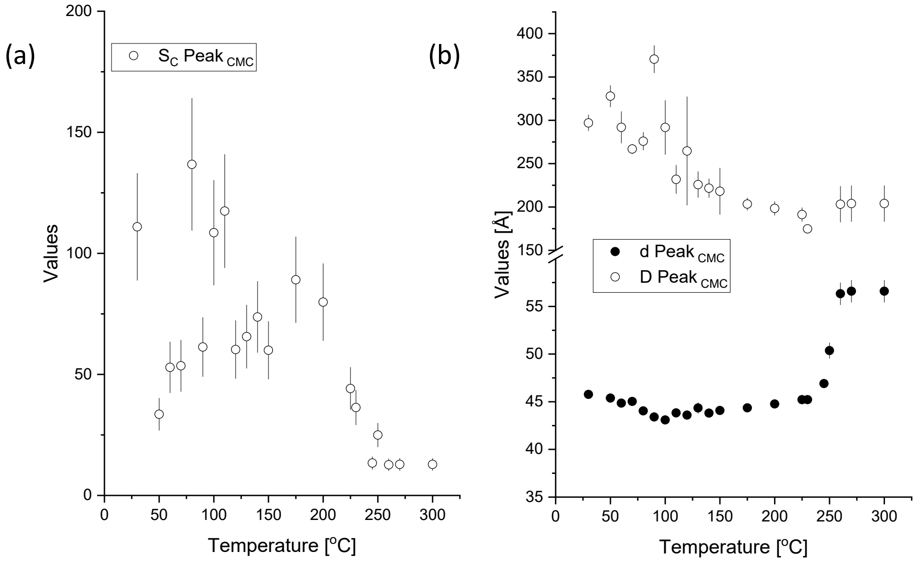

Figure S38 – Results for the modeling of X-ray scattering data. (a) scale factor for peak contribution of CMC region. (b) periodicity and domain size for CMC region. For details, see article text.

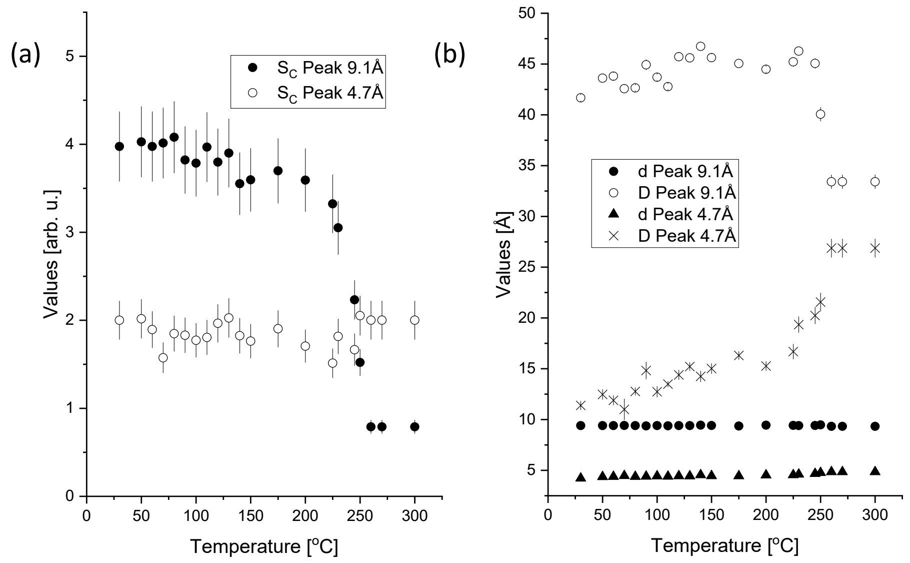

Figure S39 – Results for the modeling of X-ray scattering data. (a) scale factor for the peaks of 9Å and 4Å at WAXS region. (b) periodicity and domain size for the peaks of 9Å and 4Å at WAXS region. For details, see article text.

d. Bleached hair – Meridional cuts

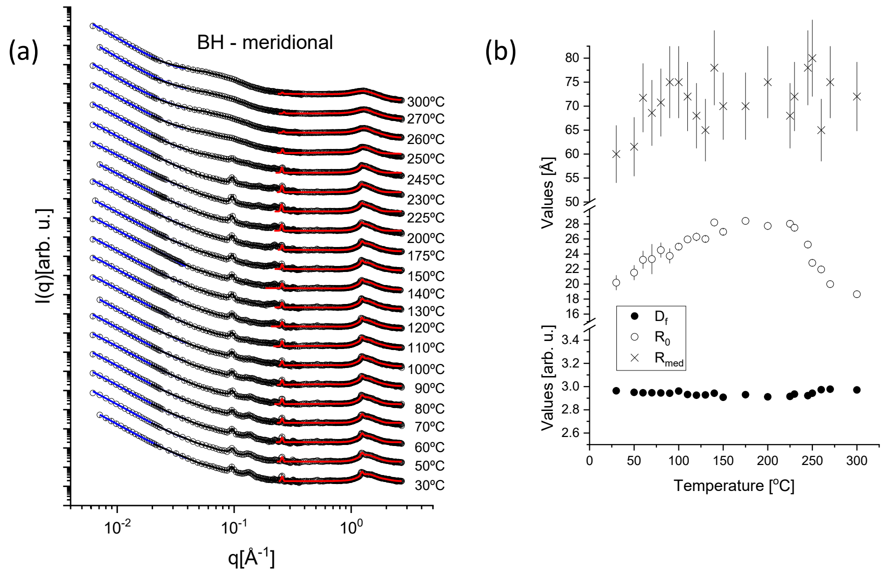

Figure S40 – Results for the modeling of X-ray scattering data. (a) X-ray scattering curves for each temperature (symbols) and theoretical curves (solid lines). Blue curves: USAXS fit, black curves: SAXS fit, red curves: WAXS fits. (b) results for the USAXS fit. For details, see article text.

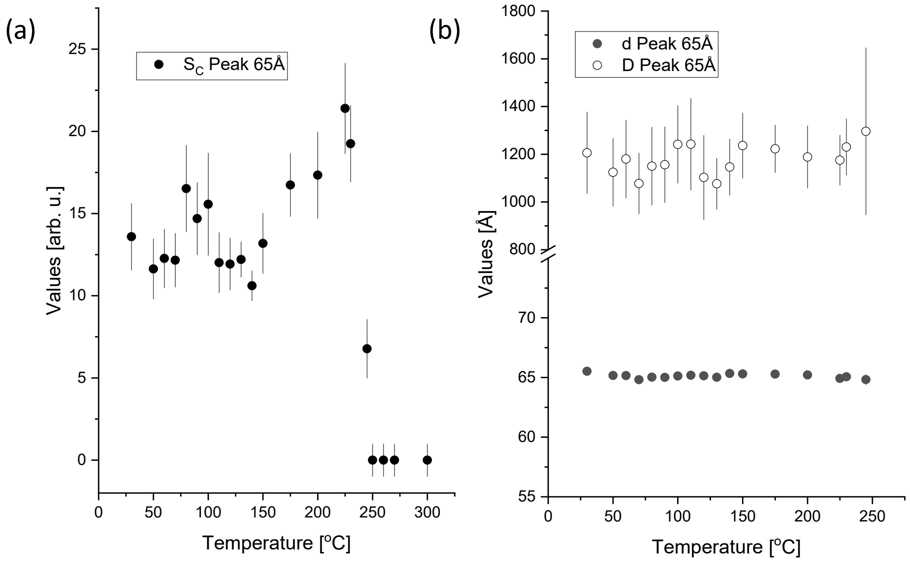

Figure S41 – Results for the modeling of X-ray scattering data. (a) scale factor for the peak of 65Å at SAXS region. (b) periodicity and domain size for the peak of 65Å. For details, see article text.

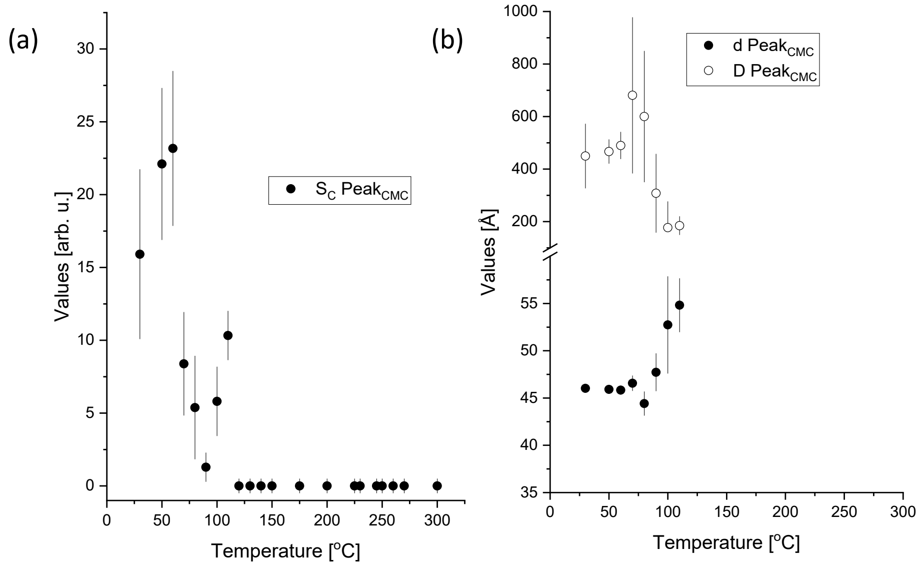

Figure S42 – Results for the modeling of X-ray scattering data. (a) scale factor for the peak of 45Å at SAXS region. (b) periodicity and domain size for the peak of 45Å. For details, see article text.

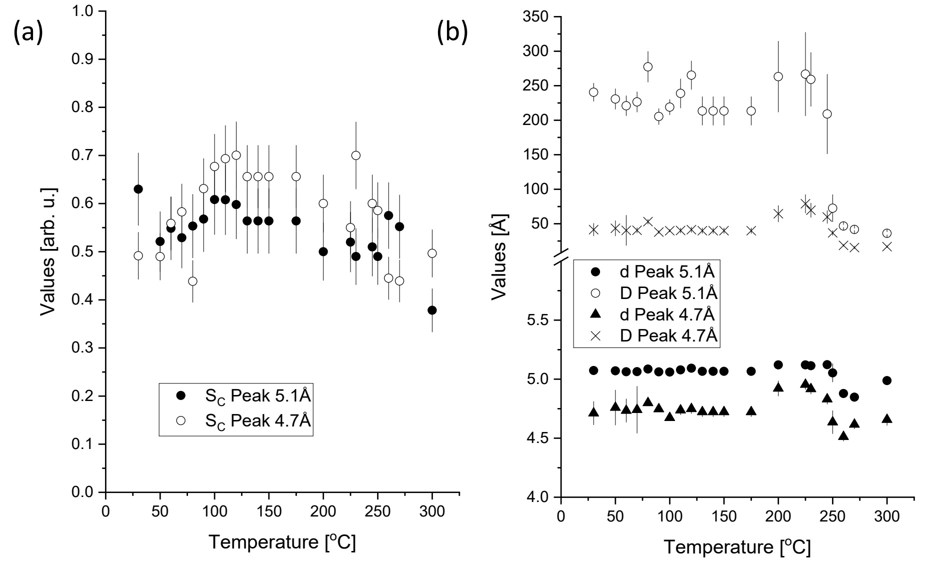

Figure S43 – Results for the modeling of X-ray scattering data. (a) scale factor for the peaks of 5.1Å and 4.7Å at WAXS region. (b) periodicity and domain size for the peaks of 5.1Å and 4.7Å at WAXS region. For details, see article text.

e. Straightened hair – equatorial cuts

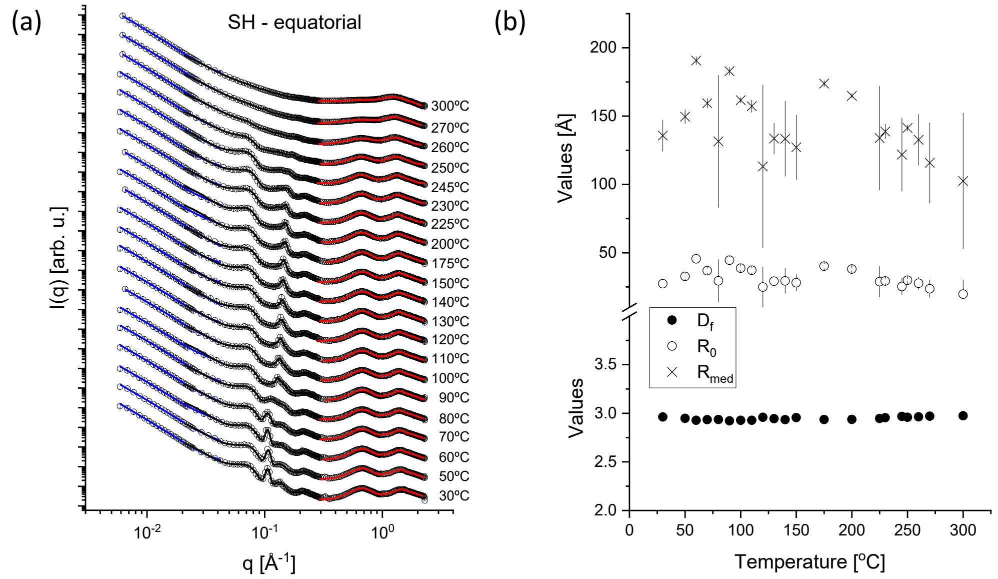

Figure S44 – Results for the modeling of X-ray scattering data. (a) X-ray scattering curves for each temperature (symbols) and theoretical curves (solid lines). Blue curves: USAXS fit, black curves: SAXS fit, red curves: WAXS fits. (b) results for the USAXS fit. For details, see article text.

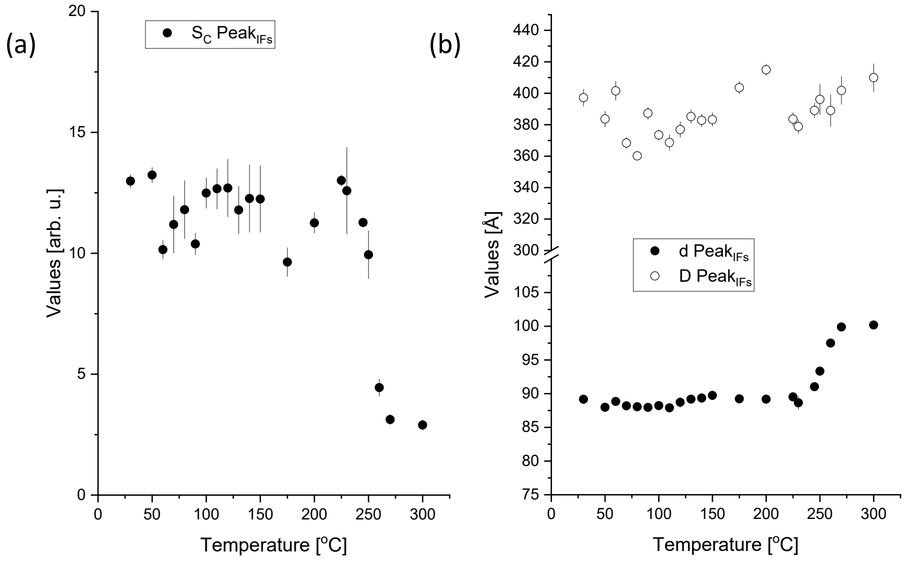

Figure S45 – Results for the modeling of X-ray scattering data. (a) scale factor for peak contribution of IFs region. (b) periodicity and domain size for IFs region. For details, see article text.

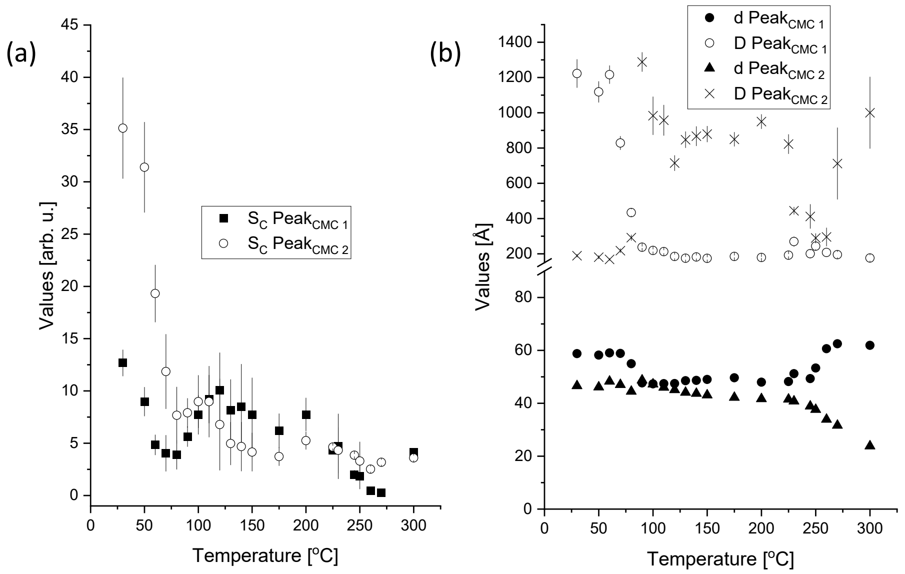

Figure S46 – Results for the modeling of X-ray scattering data. (a) scale factor for peaks contribution of CMC region. (b) periodicity and domain size for peaks at CMC region. For this case, one has two peaks at this region. For details, see article text.

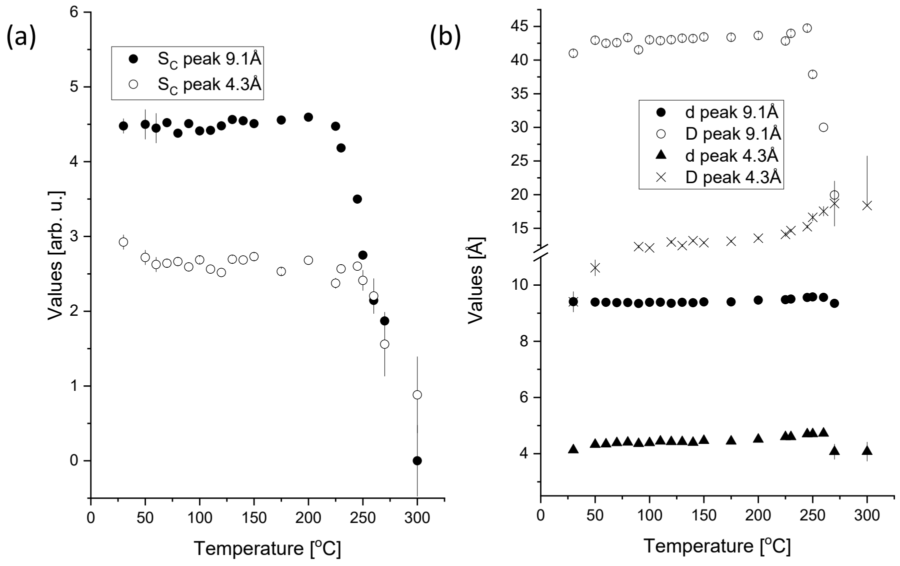

Figure S47 – Results for the modeling of X-ray scattering data. (a) scale factor for the peaks of 9Å and 4Å at WAXS region. (b) periodicity and domain size for the peaks of 9Å and 4Å at WAXS region. For details, see article text.

f. Straightened hair – Meridional cuts

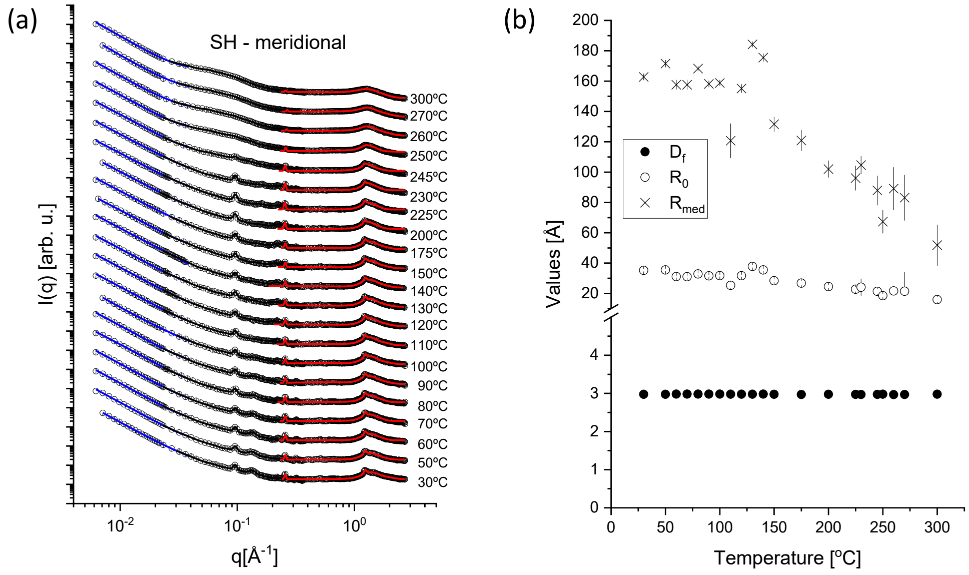

Figure S48 – Results for the modeling of X-ray scattering data. (a) X-ray scattering curves for each temperature (symbols) and theoretical curves (solid lines). Blue curves: USAXS fit, black curves: SAXS fit, red curves: WAXS fits. (b) results for the USAXS fit. For details, see article text.

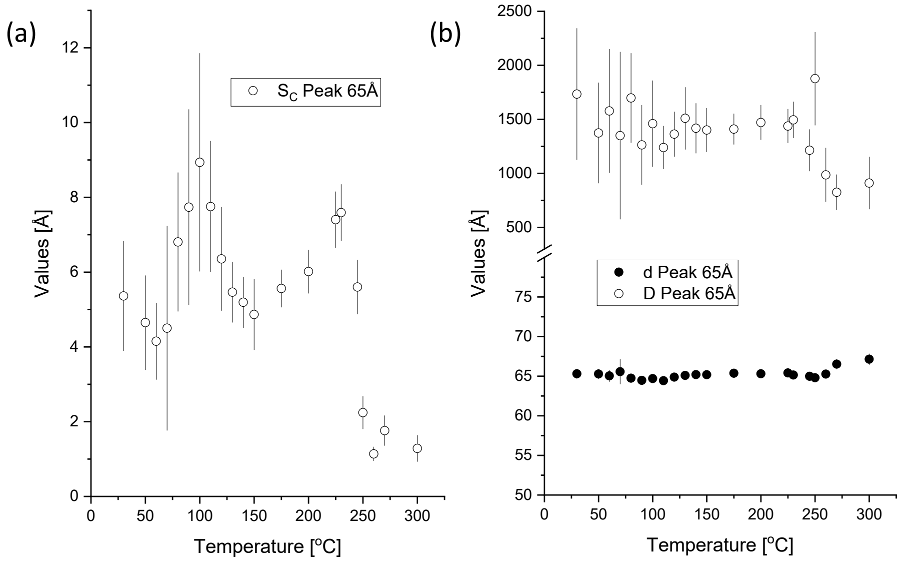

Figure S49 – Results for the modeling of X-ray scattering data. (a) scale factor for the peak of 65Å at SAXS region. (b) periodicity and domain size for the peak of 65Å. For details, see article text.

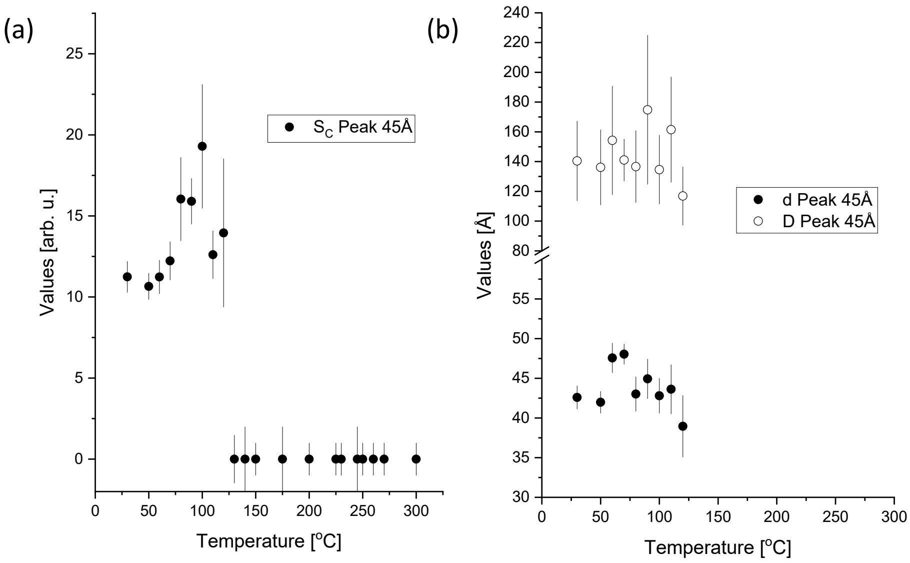

Figure S50 – Results for the modeling of X-ray scattering data. (a) scale factor for the peak of 45Å at SAXS region. (b) periodicity and domain size for the peak of 45Å. For details, see article text.

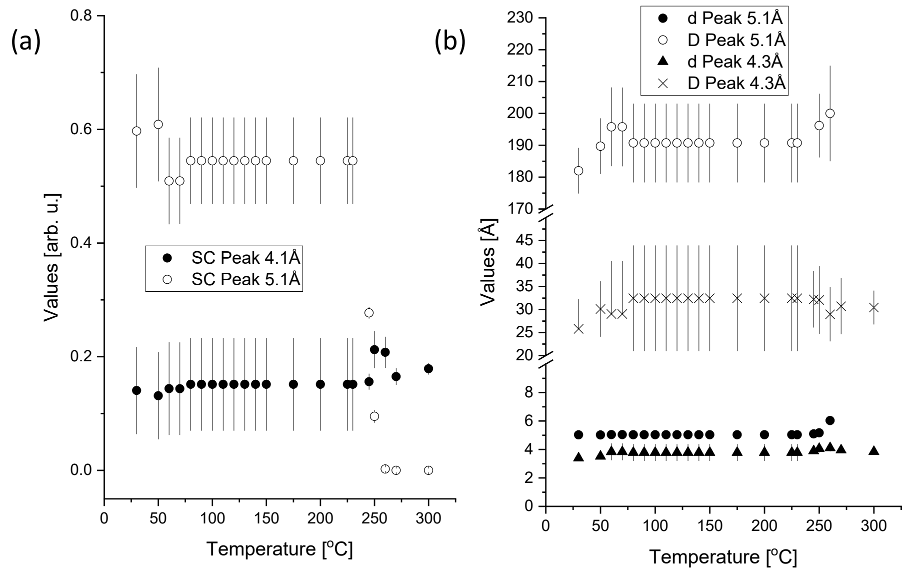

Figure S51 – Results for the modeling of X-ray scattering data. (a) scale factor for the peaks of 5.1Å at WAXS region. (b) periodicity and domain size for the peaks of 5.1Å WAXS region. For details, see article text.

g. Bleached and Straightened hair – equatorial cuts

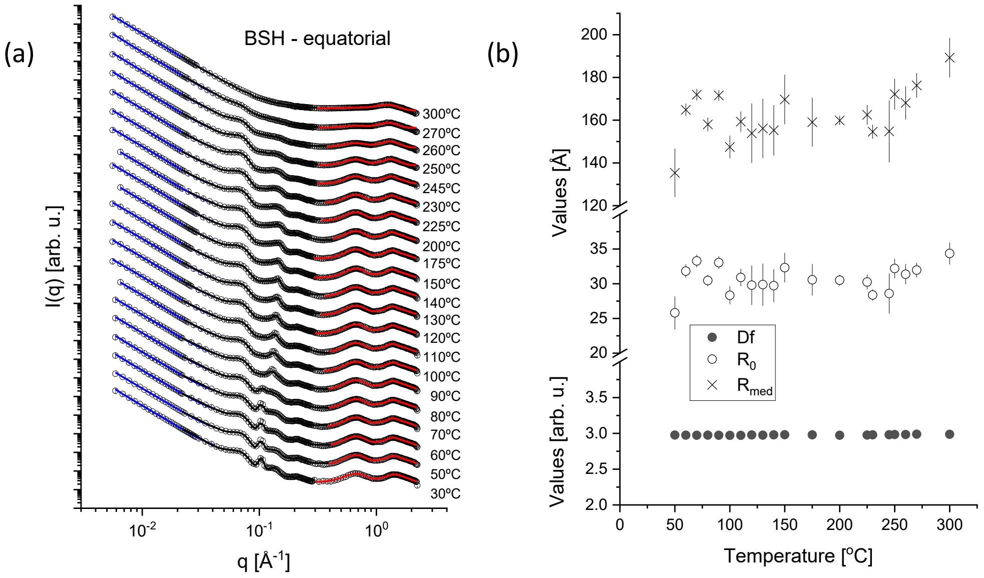

Figure S52 – Results for the modeling of X-ray scattering data. (a) X-ray scattering curves for each temperature (symbols) and theoretical curves (solid lines). Blue curves: USAXS fit, black curves: SAXS fit, red curves: WAXS fits. (b) results for the USAXS fit. For details, see article text.

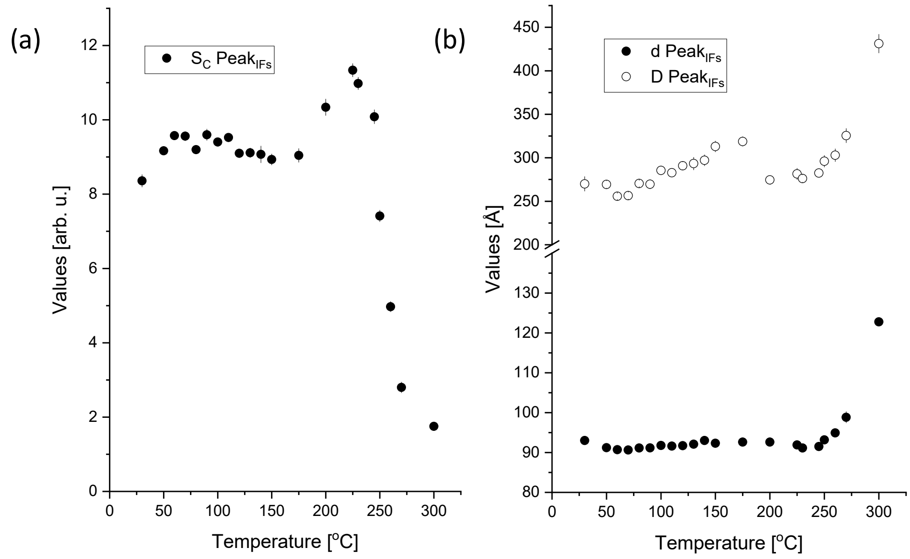

Figure S53 – Results for the modeling of X-ray scattering data. (a) scale factor for peak contribution of IFs region. (b) periodicity and domain size for IFs region. For details, see article text.

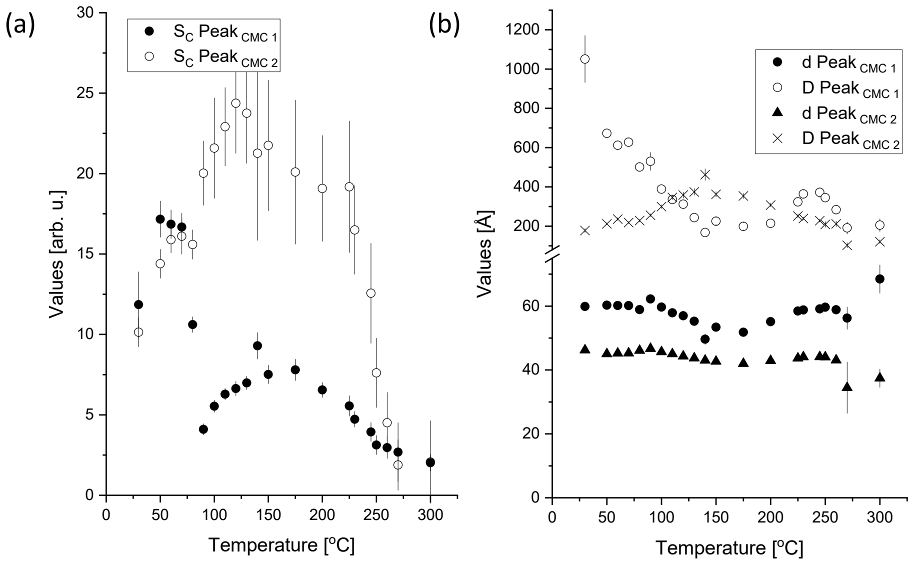

Figure S54 – Results for the modeling of X-ray scattering data. (a) scale factor for peaks contribution of CMC region. (b) periodicity and domain size for peaks at CMC region. For this case, one has two peaks at this region. For details, see article text.

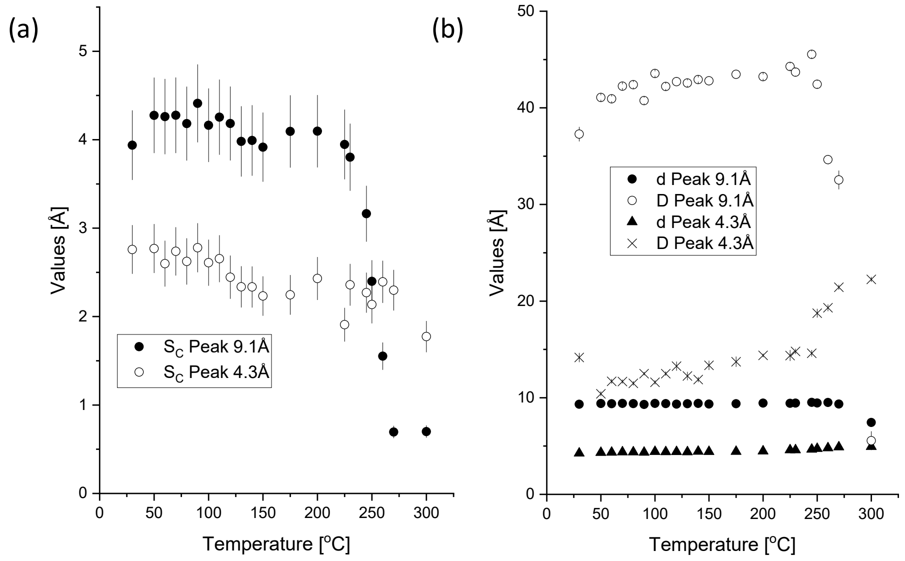

Figure S55 – Results for the modeling of X-ray scattering data. (a) scale factor for the peaks of 9Å and 4Å at WAXS region. (b) periodicity and domain size for the peaks of 9Å and 4Å at WAXS region. For details, see article text.

h. Bleached and Straightened hair – Medirional cuts

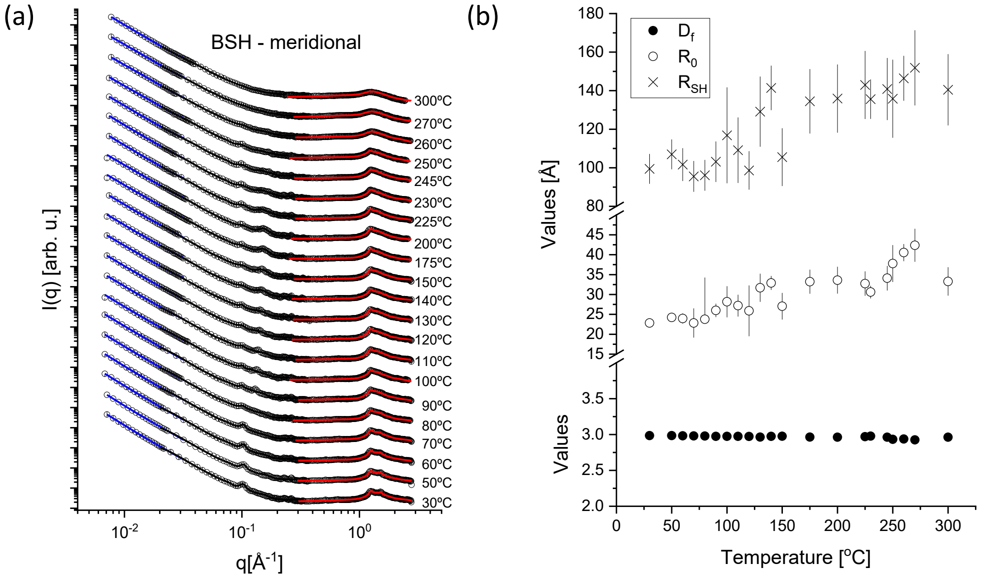

Figure S56 – Results for the modeling of X-ray scattering data. (a) X-ray scattering curves for each temperature (symbols) and theoretical curves (solid lines). Blue curves: USAXS fit, black curves: SAXS fit, red curves: WAXS fits. (b) results for the USAXS fit. For details, see article text.

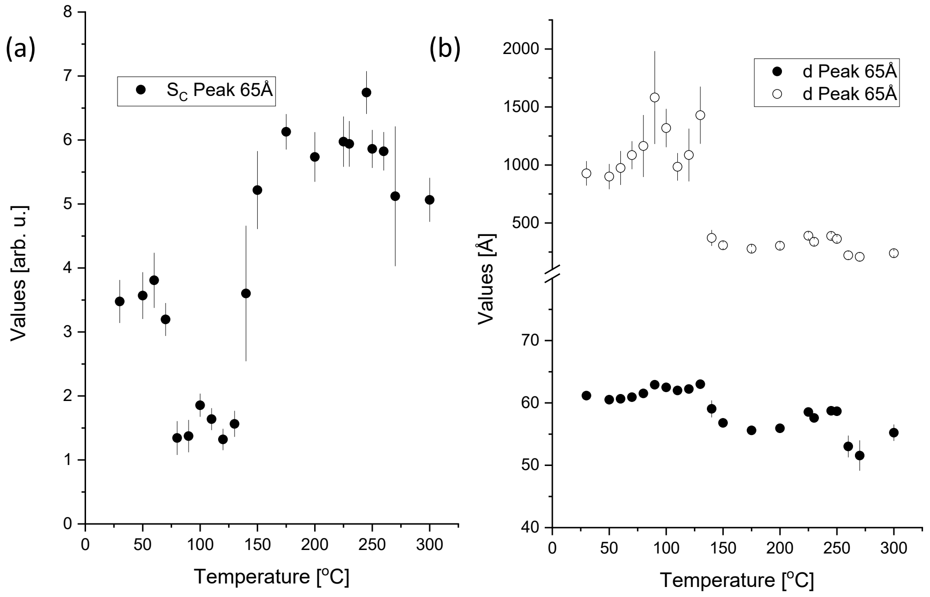

Figure S57 – Results for the modeling of X-ray scattering data. (a) scale factor for the peak of 65Å at SAXS region. (b) periodicity and domain size for the peak of 65Å. For details, see article text.

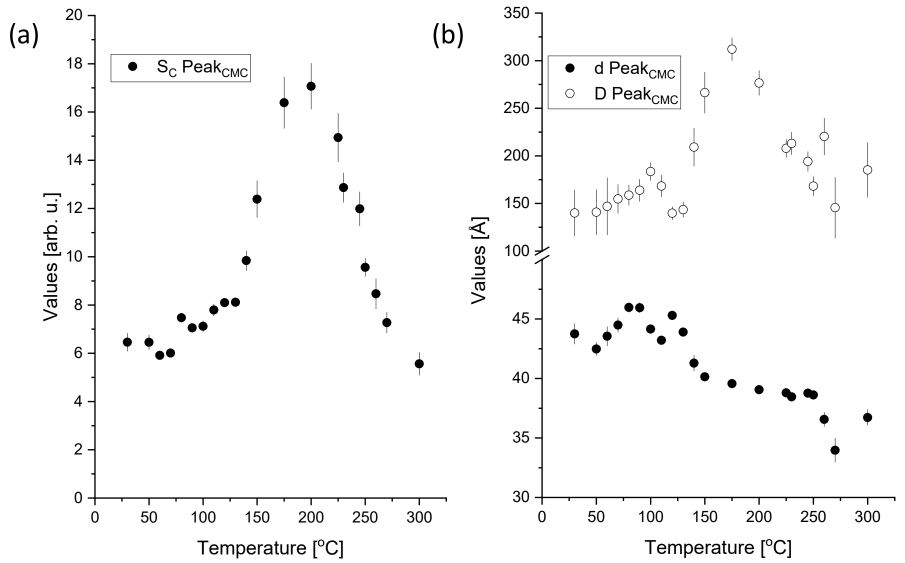

Figure S58 – Results for the modeling of X-ray scattering data. (a) scale factor for the peak of 45Å at SAXS region. (b) periodicity and domain size for the peak of 45Å. For details, see article text.

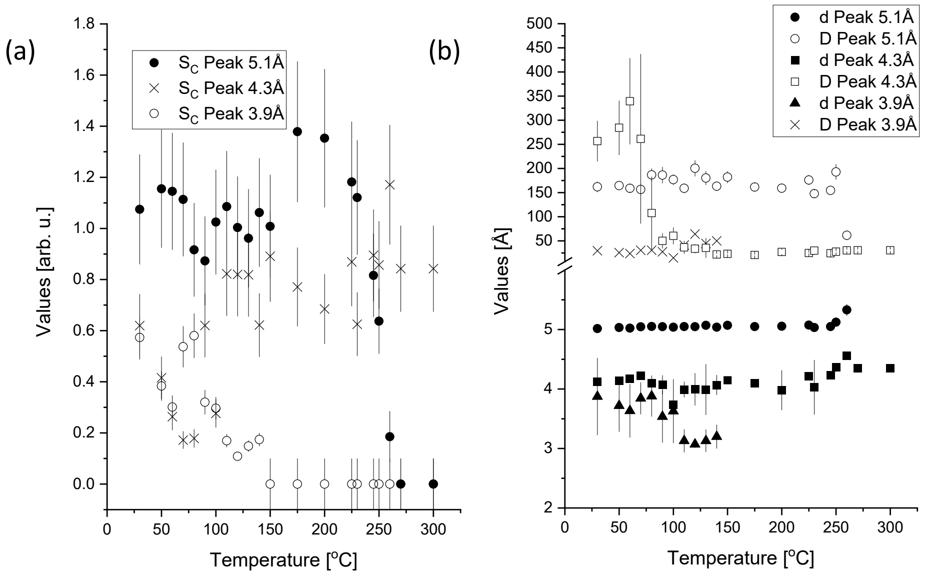

Figure S59 – Results for the modeling of X-ray scattering data. (a) scale factor for the peaks of 5.1, 4.3 and 3.9 Å at WAXS region. (b) the peaks of 5.1, 4.3 and 3.9 Å at WAXS region. The peak of 3.9Å disappears after 150°C. For details, see article text.
